# Kinase Inhibitors can Change Protonation or Tautomeric State upon Binding

**DOI:** 10.1021/acs.jcim.6c02792

**Published:** 2026-10-16

**Authors:** Gehan A. Ranepura, Saqibul I. Chowdhury, Elizabeth A. Rosenzweig, Ariën S. Rustenburg, Raquel López-Ríos de Castro, Junjun Mao, John D. Chodera, Sukrit Singh, M. R. Gunner

**Affiliations:** † Ph.D. Program in Physics, The Graduate Center, 2009 City University of New York, New York, New York 10016, United States; ‡ Department of Physics, City College of New York, New York, New York 10031, United States; § Computational and Systems Biology Program, Sloan Kettering Institute, Memorial Sloan Kettering Cancer Center, New York, New York 10065, United States; ∥ Graduate Program in Physiology, Biophysics, and Systems Biology, Weill Cornell Medical College, New York, New York 10065, United States; ⊥ Department of Physics, Freie Universität Berlin, Arnimallee 14, Berlin 14195, Germany; # Benjamin Levich Institute for Physico-Chemical Hydrodynamics, City College of New York, New York, New York 10031, United States

## Abstract

The binding affinity of a ligand to a protein is influenced by the protonation and tautomeric states of both partners. However, this relationship remains under-investigated due to the limited availability of computational tools capable of treating charge and tautomer states together in a scalable manner to study clinically relevant systems. Here, we use multi-conformation continuum electrostatics (MCCE) to calculate the protonation and tautomer distributions of nine kinase domains bound to 18 FDA-approved inhibitors across 37 cocrystal structures, while considering their Boltzmann-ensemble. The full pipeline was repeated in triplicate, so that every reported value carries a measured run-to-run uncertainty. Our simulations show that protein net charge and proton distribution remain largely stable even upon binding charged inhibitors. Our results find that individual inhibitor charges are dynamic, frequently increasing or decreasing upon binding a specific protein target. We observe that kinase-inhibitor binding shifts the relative probabilities of low-energy states (Δ*G* < 2.8 kcal/mol), but does not increase the probability of higher-energy states in the bound form. By considering all low-energy charge states and tautomers, we identify when two solution-state tautomers of nearly equal population resolve upon binding into a single state, creating free energy differences of up to 3.5 kcal/mol between tautomers, and in some complexes differences beyond the resolution of the sampling. In turn, we find that minority species can become the dominant component in the bound state, emphasizing the necessity of considering ensemble-wide protonation and tautomer states to accurately predict protein–ligand binding energetics.

## Graphical abstract

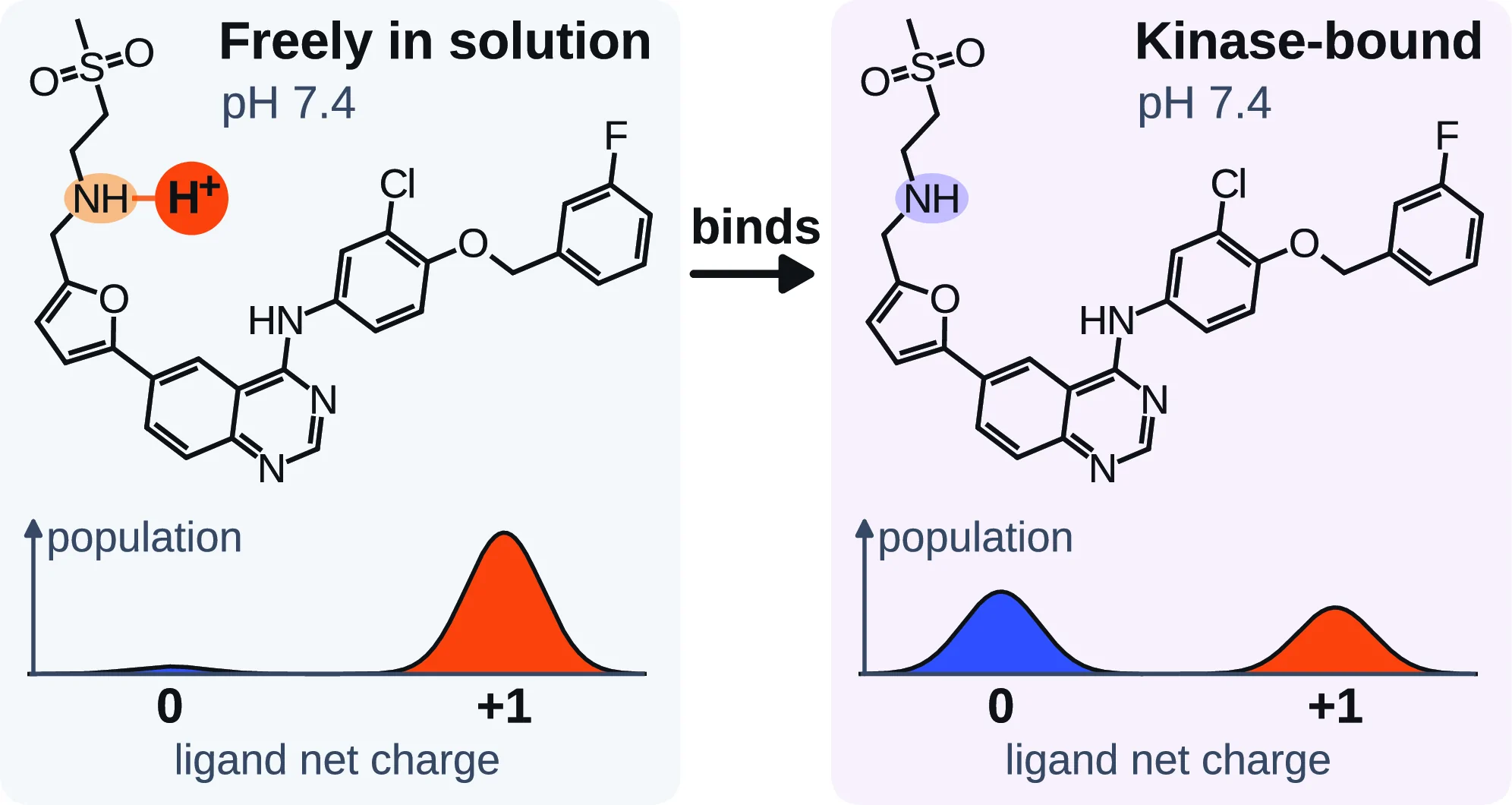

## Introduction

Computer-aided drug design (CADD) accelerates medicinal chemistry design cycles by optimizing compounds in silico before experimental testing. Drug discovery has many successful uses that involve computations incorporating protein structural information with physics-based ligand interaction energy calculations.^1^ CADD-based approaches have driven the discovery of many novel ligands and binders across a variety of biological systems.^2^ Most CADD approaches have enabled the greatest acceleration by rapidly predicting the affinity of ligands against targets and pockets of interest, estimating the free energy of inhibitor binding (Δ*G*), providing a testable measurement for rank-ordering compounds.^3^

Despite its success, free energy estimation approaches have plenty of room for improvement to estimate accurate binding energies with useful precision. Calculating the correct Δ*G* of ligand binding requires accounting for dynamic components such as the distribution of possible ligand conformations and the ensemble of possible protonation states.^4^ Consistent with induced-fit mechanisms, the apo (ligand-unbound) protein undergoes conformational rearrangement upon ligand binding to form the holo (ligand-bound) protein. This transition shifts the system’s ensemble, resulting in measurable changes in the internal enthalpy and entropy compared to the unbound state.^5^ Waters may be freed from the protein binding site, contributing to the system Δ*G*.^6^ Furthermore, the ligand itself has interactions with the solvent which are lost upon binding.^7^

One challenging property to model is the ionization state of both the ligand and the target binding site, before and after binding at physiological pH.^8–11^ At physiological pH, a protein is thought to have an average of 25% charged residues: aspartate (Asp), glutamate (Glu), arginine (Arg), and lysine (Lys).^12^ Histidine (His) may undergo an equilibrium between charge species and tautomer states at physiological pH, existing in an ensemble of multiple ionization states. This range of protein charge and tautomer states exists in tandem with the range of ligand charge and tautomer states. Ligand protonatable substituents and tautomeric states underlie an equilibrium ensemble of states that can change on binding. Changes in residue proton affinity (p*K*_a_) also occur after binding of the ligand to its target, which can shift the distribution of net charges to a new equilibrium.^13^ Many of these details are missed in standard simulation workflows. Protons are usually positioned heuristically in the protein–ligand structure, and protonation states are held fixed throughout simulations. As a result, protonation and tautomeric states can be misassigned, causing large errors in drug-binding affinity predictions.^14,15^ Most free energy estimations, and equilibrium molecular simulation methods, constrain the protonation state and net charge of a system to reduce computational cost and the degree of sampling required.

Computing protein and ligand protonation states, and their changes upon binding, has demonstrated relevance to cellular signaling and drug design.^16,17^ For example, tyrosine kinases are a major class of drug targets, since they are implicated in 1/3 of oncogenic mutations^4^ as well as the etiology of several inflammatory disorders such as rheumatoid arthritis and psoriasis.^18^ Kinase inhibitors are likewise an important class of useful compounds, and there are a significant number of protein structures which have been crystallized with FDA-approved inhibitors.^19^ Previous work has shown the pH dependence of kinases on both the activation process,^20^ the inactivation of kinases in cellular contexts,^17^ and their importance for drug binding. Thus, to accurately predict-ligand binding affinities, functionally relevant protein ensembles, and the sensitivity of ligand and protein net charge to drug binding, we must consider the full distribution of protons in both targets and ligands. Protonation states, tautomeric states, or proton positions are all required for reliable downstream physics-based modeling. Even high-quality experimental or predicted structures do not provide proton positions or the protonation states of acidic or basic groups. Experimental approaches such as multidimensional NMR can provide highly accurate information about residue or ligand protonation state, but are generally not compatible with high-throughput drug-discovery workflows and have high sample requirements.

Therefore, there is a clear need for an efficient methodology to compute protonation and tautomer populations in protein–ligand complexes. Faster computational p*K*_*a*_-prediction tools, such as PROPKA,^21^ are useful, but often require additional heuristics or user expertise to translate predicted p*K*_a_ shifts into concrete protonation, tautomer, and proton-position assignments for simulation. Some simulation methods have emerged that enable the tracking of conformational, tautomeric, and protonation states such as constant-pH MD and Grand Canonical Monte Carlo Methods (GCMC).^22,23^ However, GCMC methods are computationally efficient at the cost of sacrificing a full exploration of conformational space found in MD. Conversely, constant-pH MD (cpH-MD) requires extensive sampling at time-frames that are too slow for modern drug design pipelines.^2,22^

Computational methods based on Monte Carlo sampling using a continuum electrostatics (CE) force field offer an efficient approach to rapidly compute charge states and electrostatic interactions that control protonation states.^24,25^ CE is a physics-based coarse-grained model of environmental polarization that instantaneously equilibrates the solvent response to charge. Unlike standard equilibrium MD, which typically uses a background dielectric constant of 1 by default to account for electronic polarization, CE incorporates homogeneous electronic polarizability through an effective dielectric constant treatment, typically using dielectric constant values of 4 within the protein and 80 for the surrounding solvent. GCMC methods are also able to consider explicit buried waters in addition to the surrounding continuum solvent, side chain conformations, and ligand configurational changes as relevant.^24^

In this work, we use a GCMC-based CE method, called Multi-Conformation Continuum Electrostatics (MCCE),^26^ to calculate the distribution of inhibitor protonation/tautomer states in solution versus when bound to the protein by combining molecular mechanics and CE energy terms. MCCE samples ligand and protein tautomer and protonation states as well as protein side chain rotamers to calculate the Boltzmann distribution of conformers of ligands and side chains (see Methods).

We compare the Boltzmann distributions of protonation and tautomer states for the apoprotein and solvated ligand against the ligand-bound complex to determine the shift in distribution upon inhibitor-binding. The relative affinity of individual inhibitor states is assessed via GCMC, where all inhibitor species are allowed to reach equilibrium, partitioning between the bound and free states according to their respective chemical potentials.^26–32^

We scale our MCCE-based pipeline to pharmacologically relevant targets: namely protein tyrosine and serine/threonine kinases and known FDA-approved inhibitors. We consider 37 experimental structures of 18 unique inhibitors cocrystallized with 9 unique kinase domains spanning ABL, ALK, CDK6, DDR1, EGFR, JAK3, MEK, MET, and VEGFR. Ponatinib occupies two distinct sites in 3ZOS, a buried active site and a solvent-exposed surface site, so the 37 structures yield 38 inhibitor binding sites; ligand quantities are reported per site and protein quantities per structure. We find that these kinases have minor changes in net charge upon ligand binding even when binding a charged inhibitor. Some inhibitors become less charged upon binding, as expected given the loss of solvation energy as they move out of water. However, other binding pockets prefer a charged ligand, leading to a higher ligand charge when bound. Further, we identify some inhibitors that favor one tautomeric state in solution and a different one when bound. We also test whether these charge changes track the kinase conformational state, and find no clear dependence: DFG-in structures show a broader spread of inhibitor charge change than DFG-out structures, but this reflects only a subset of DFG-in ligands, so the conformational label is not predictive of the degree of charge change in either partner. Taken together, we find that many inhibitor protonation and tautomeric states upon binding cannot be extrapolated from the solution state alone, and must be considered in-complex. Our findings have important implications for drug discovery pipelines, as they highlight the need to consider the protonation state of candidate molecules in both the solvated and target-bound forms separately for an accurate pipeline.

## Methods

### Software and Computational Environment

All calculations were performed using the following software versions: Schrödinger Suite (Epik, version 2017–2),^33,34^ OpenEye Toolkit v2017.Jun.1 (OEChem, Omega, AM1-BCC charging module), and Python 3.6 for workflow orchestration. Epik and OpenEye calculations were performed on Microsoft Windows 10. The MCCE program (Multi-Conformation Continuum Electrostatics, version 4) was used for subsequent electrostatic p*K*_a_ calculations and protonation state population analysis of protein-inhibitor complexes on Linux.^26,29,35^

### Input Structure Preparation

Nine different kinase types with 18 FDA-approved inhibitors, across 37 cocrystal structures, were investigated (Figure S1). X-ray crystal structures were obtained from the RCSB Protein Data Bank, each comprising a single kinase domain. The protein regions included in the calculations are listed in Table S1. The deposited coordinates were used as supplied, with no truncation, capping or loop repair, so the resolved extent of the construct differs between entries, from 260 residues in 5L2I to 526 in 2EUF, which is the one entry with two chains. This bears on how the protein charges of Table 1 should be read: an apo or holo charge is a sum over the ionizable residues present in that particular model, so absolute charges are not comparable between structures that resolve different portions of the construct. The apo-to-holo difference is taken within a single structure and is unaffected, and indeed shows no relationship to construct size across the set (*r* = 0.01 between the number of ionizable residues and Δcrg_Holo‑Apo_). Crystallographic water molecules were removed and replaced with implicit solvent modeled with a dielectric constant of 80 within the MCCE simulation. The 18 small molecule kinase inhibitors were parametrized for MCCE calculations using a combined workflow of protonation state enumeration via Schrödinger Epik partial charge assignment via the OpenEye Toolkit with the AM1-BCC charge model.

**Table 1:** Charges of Isolated Ligands, Apo-Kinase and changes on Binding, Averaged Over Three Independent Repeats^a^

PDBID	Ligand	#conf	crg_soln_	crg_bound_	Δcrg_Lig_	ΔTaut	crg_Apo_	crg_Holo_	Δcrg_Holo‑Apo_	DFG
ABL: ABL1 Tyrosine Protein Kinase										
3UE4	Bosutinib	4	0.94	0.95	+0.01	yes	–4.58	–4.89	–0.31	in
2HYY	Imatinib	5	0.97	1.00	+0.03	yes	–6.19	–6.37	–0.18	out
3PYY	Imatinib	5	0.97	1.00	+0.03	yes	–4.81	–4.85	–0.04	out
3CS9	Nilotinib	3	0.09	0.41	+0.32	no	–4.60	–4.63	–0.02	out
5MO4	Nilotinib	3	0.09	0.24	+0.15	no	–7.94	–7.95	–0.01	out
ALK: Anaplastic Lymphoma Kinase										
3AOX	Alectinib	2	0.56	0.83	+0.27	N/A	–8.38	–8.42	–0.04	in
4MKC	Ceritinib	3	1.00	1.00	0.00	N/A	–4.06	–4.65	–0.59	in
2XP2	Crizotinib	4	1.61	1.00	–0.61	no	–5.80	–5.86	–0.06	in
2YFX	Crizotinib	4	1.61	1.00	–0.61	no	–4.59	–4.61	–0.02	in
4ANQ	Crizotinib	4	1.61	1.00	–0.61	no	–5.61	–5.66	–0.05	in
4ANS	Crizotinib	4	1.61	1.00	–0.61	no	–5.73	–5.78	–0.05	in
5AAA	Crizotinib	4	1.61	1.00	–0.61	no	–5.43	–5.48	–0.05	in
5AAB	Crizotinib	4	1.61	1.00	–0.61	no	–6.22	–6.28	–0.06	in
5AAC	Crizotinib	4	1.61	1.00	–0.61	no	–5.63	–5.71	–0.08	in
CDK6: Cyclin-Dependent Kinase 6										
2EUF	Palbociclib	2	0.98	1.00	+0.02	N/A	–9.59	–9.76	–0.17	in
5L2I	Palbociclib	2	0.98	0.99	+0.01	N/A	–0.23	–0.21	0.02	in
DDR1: Discoidin Domain Receptor Tyrosine Kinase										
3ZOS(b)	Ponatinib	3	0.96	1.00	+0.04	yes	–1.65	–1.69	–0.04	out
3ZOS(s)	Ponatinib	3	0.96	0.90	–0.06	yes	—	—	—	out
EGFR: Epidermal Growth Factor Receptor Kinase										
4G5J	Afatinib	3	0.99	0.95	–0.04	N/A	–6.47	–6.60	–0.13	in
4G5P	Afatinib	3	0.99	0.96	–0.03	N/A	–0.61	–0.93	–0.32	in
2ITO	Gefitinib	5	0.30	0.78	+0.48	no	–7.08	–7.26	–0.18	in
2ITY	Gefitinib	5	0.30	0.35	+0.05	no	–6.03	–6.11	–0.08	in
2ITZ	Gefitinib	5	0.30	0.65	+0.35	no	–4.87	–4.98	–0.10	in
3UG2	Gefitinib	5	0.30	0.01	–0.29	no	–3.01	–2.95	0.06	in
4I22	Gefitinib	5	0.30	0.50	+0.20	no	1.92	1.85	–0.07	in
4WKQ	Gefitinib	5	0.30	0.46	+0.16	no	–1.35	–1.36	–0.01	in
1XKK	Lapatinib	3	0.95	0.58	–0.37	N/A	0.48	0.09	–0.39	in
4ZAU	Osimertinib	7	0.98	0.99	+0.01	no	–2.13	–2.44	–0.31	in
JAK3: Janus Kinase 3										
3LXK	Tofacitinib	2	0.03	0.05	+0.02	N/A	1.18	1.26	0.08	in
MEK: Mitogen-Activated Protein Kinase										
4AN2	Cobimetinib	3	0.99	0.22	–0.77	no	–3.52	–3.66	–0.15	in
4LMN	Cobimetinib	3	0.99	0.52	–0.47	no	–3.57	–3.58	–0.01	in
MET: Tyrosine Protein Kinase Met										
2WGJ	Crizotinib	4	1.61	0.96	–0.65	no	7.02	6.78	–0.24	in
VEGFR: Vascular Endothelial Growth Factor Receptor Kinase										
4AG8	Axitinib	3	0.00	0.00	0.00	yes	4.58	4.61	0.03	out
4AGC	Axitinib	3	0.00	0.00	0.00	yes	3.49	3.50	0.01	out
3WZD	Lenvatinib	3	0.00	0.00	0.00	no	1.56	1.58	0.02	in
3WZE	Sorafenib	4	0.00	0.00	0.00	no	5.51	5.50	–0.01	out
4ASD	Sorafenib	4	0.00	0.00	0.00	no	0.22	0.22	0.00	out
4AGD	Sunitinib	4	0.99	0.90	–0.09	no	2.53	2.44	–0.09	out

a Results grouped by kinase type. Calculations carried out on “raw” PDB files. Every value is the mean over Trial01–03; the per-trial values, their means and their standard errors are given in Table S4. Reported in this table are the number of protonation/tautomer states within 6 kcal/mol of the lowest conformer (#conf), which is a property of the ligand and does not vary between trials; the Boltzmann weighted ligand charge in solution (crg_soln_); the MCCE calculated charge of the ligand bound in the PDB structure (crg_bound_); the change from crg_soln_ to crg_bound_ (Δcrg_Lig_); the MCCE calculated charges of the kinase residues with (crg_Holo_) and without (crg_Apo_) the inhibitor bound, neither of which includes the ligand charge; and the change in protein charge upon binding (Δcrg_Holo‑Apo_), computed within each trial before averaging, so it can differ from the difference of the two rounded means by 0.01. All ligand calculations account for the relative Epik state penalty Δ*G*_total_ defined in the Methods. Ponatinib occupies two sites in 3ZOS, buried (b) and surface (s); the protein columns are given once. DFG: Orientation of DFG loop (in–active; out–inactive).

### Protonation State Prediction

The initial three-dimensional coordinates for each inhibitor were obtained from the RCSB protein data bank (PDB) crystallographic structure files in MOL2, PDB, or SDF format. The chemically relevant inhibitor conformer proton positions, atomic charges, and state penalties were enumerated at physiological pH (7.4) using Epik from the Schrödinger Suite (version 2017-2).^33,34^ The input molecules in MOL2 format were converted to Maestro (MAE) format and processed with Epik using the following command–line parameters: pH = 7.4, pH tolerance of 10.0, 50 maximum structures, no tautomer enumeration, and calculation of atomic p*K*_a_ values. A maximum energy penalty threshold of 10.0 kT was applied, corresponding to a minimum state probability of exp(−10) ≈ 4.54 × 10^–5^ at 298 *K*, to exclude energetically unfavorable microstates while retaining all chemically significant protonation states.

For each generated state, Epik computed a total state penalty (Δ*G*_total_) decomposed into its ionization and tautomer components quantifying the relative cost of free energy in kcal/mol

1 $$\Delta G_{ionization}=\Delta G_{charging}+\Delta G_{neutral}$$

2 $$\Delta G_{tautomer}=\Delta G_{total}-\Delta G_{ionization}$$

In eq 1 the ionization penalty for charging (Δ*G*_charging_) represents the energetic cost of forming charged species from neutral forms, while the neutral penalty (Δ*G*_neutral_) accounts for deviations from the optimal neutral state. The tautomer component of eq 2 is what distinguishes states of equal net charge. The net formal charge for each state was extracted from the Epik output metadata.

The free energy of each inhibitor at a given pH is used to determine the p*K*_a_ relative to a reference state^36^

3 $$pK_{a}=\frac{\Delta G_{deprot}}{2.303k_{B}T}+{pH}_{ref}$$

Equation 3 gives the p*K*_a_ of a single ionizable site. Since tautomers have the same number of protons, they have uniform p*K*. Instead of using p*K*, the probability *P*(*i*) of each conformer is obtained from the Boltzmann distribution

4 $$P\left(i\right)=\frac{e^{-\Delta G_{i}/k_{B}T}}{Z}$$

where the partition function *Z* in eq 4 is the sum of the Boltzmann weights over all conformers of that inhibitor

5 $$Z=\sum_{j}e^{-\Delta G_{j}/k_{B}T}$$

### Partial Charge Assignment

Atomic partial charges were calculated for each protonation state using the semiempirical AM1-BCC (Austin model 1 with bond charge corrections) method implemented in the OpenEye Toolkit. Epik output structures in MAE format were converted to SDF, and each protonation state was processed independently. Conformational ensembles were generated using OpenEye Omega with a maximum of 800 conformers per state (max_confs_ = 800), flexible stereochemistry (strictStereo = false) and retention of all generated conformers (keep_confs_ = −1 or none) to enable the calculation of the conformationally averaged charge.

AM1-BCC charges were computed using the OpenEye get_charges­() function with charge normalization enabled to ensure the sum of partial charges equals the integer net charge determined by Epik. The AM1-BCC method combines semiempirical AM1 quantum chemical calculations with empirical bond charge corrections fitted to reproduce Hartree–Fock 6-31G* electrostatic potentials, providing an efficient and accurate charge model suitable for molecular mechanics simulations. Charges were averaged across all generated conformers to account for the effects of conformational flexibility on the electrostatic distribution.

For each successfully parametrized protonation state, charged MOL2 and PDB files with preserved atomic nomenclature were generated. Atom names from the original input structure were maintained throughout the workflow to ensure consistency with protein–ligand complex preparation. PDB files were written using OpenEye OEChem with flags enabling proper handling of current residues, element types, standard bonds, heteroatom bonds, and dual coordinate sets.

States for which the AM1-BCC charge calculation failed were saved in a separate directory with the corresponding error logs for diagnostic purposes. State penalty summaries were tabulated for all successfully processed states, documenting the state identifier, net charge, ionization penalty components, tautomer penalty, and total state penalty. Complete procedural logs recording software versions, parameters and calculation outcomes were generated for each inhibitor to ensure computational reproducibility according to the FAIR (Findable, Accessible, Interoperable, Reusable) data principles.

### MCCE Computational Workflow

For each kinase–inhibitor system, three independent MCCE calculations were performed: (1) the apoprotein, where the ligand is removed from the holoprotein structure without additional relaxation; (2) the holoprotein complex; and (3) the isolated ligand. The difference in the probability of each inhibitor protonation or tautomer state between the bound and free forms is then determined. Figure 1 describes the overall workflow.^26^

**Figure 1:**
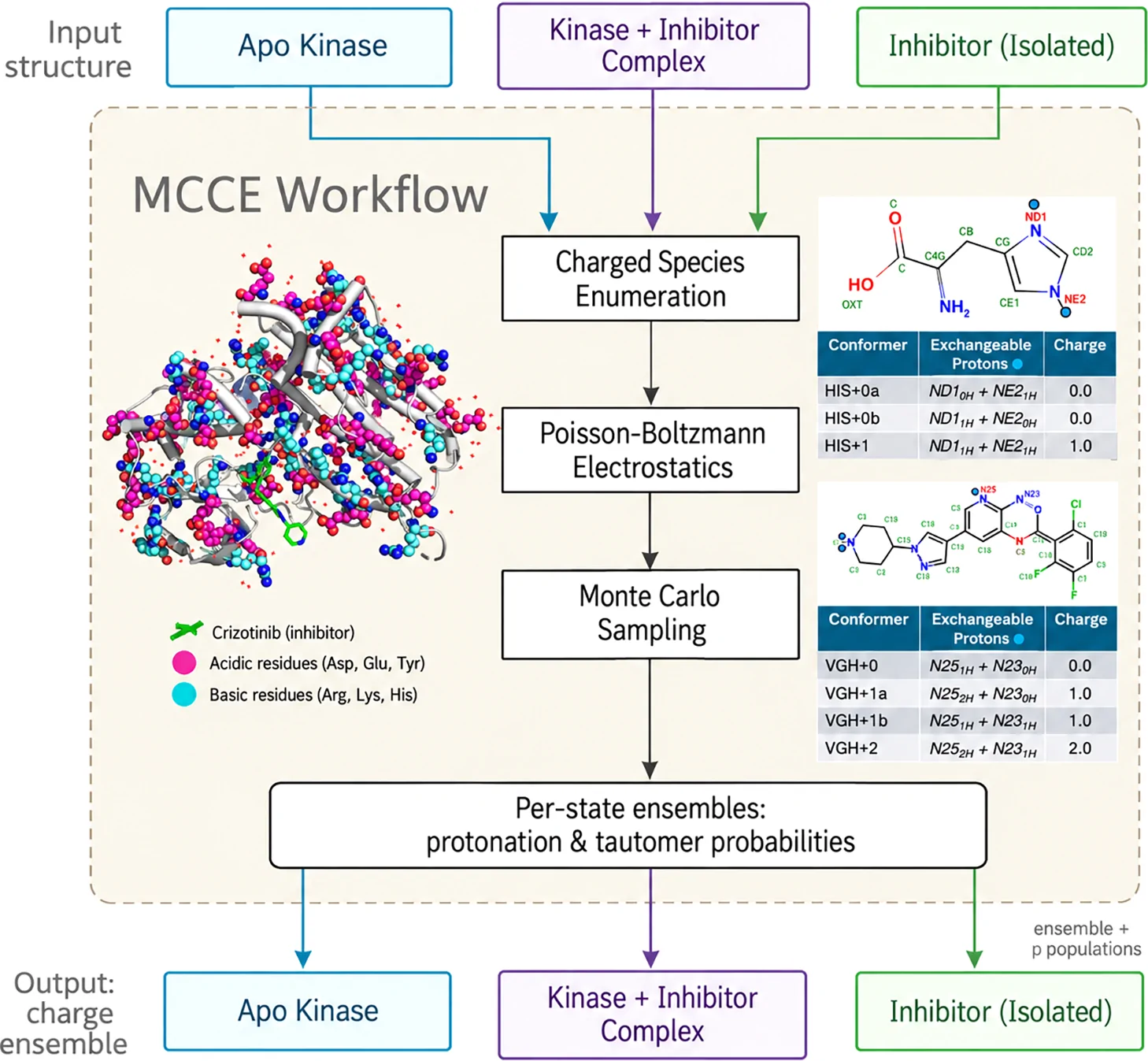
MCCE workflow. Row 1: MCCE4 is run independently on three systems, the apo kinase (inhibitor removed), the holo kinase (inhibitor bound), and the inhibitor in solution. Row 2: conformers sample protonation states (amino acids and inhibitor), tautomers (His and inhibitor), and polar proton orientations. Left panel: human anaplastic lymphoma kinase with crizotinib (green sticks; PDB ID: 2XP2), spheres marking ionizable residues whose charge state can change (acids, magenta; bases, cyan). Right panel: conformer tables for His and for crizotinib, with the other inhibitors in Figure S1. Each His has one charged (+1) and two neutral tautomers; crizotinib conformers span net charges of 0, +1 and +2, where the subscripts (0H, 1H, 2H) give the number of protons on each nitrogen and labile nitrogen labels are red. Row 3: Poisson–Boltzmann calculations build energy lookup tables of self-energies (solvation, torsion and interactions with backbone dipoles) and pairwise conformer–conformer interactions. Rows 4 and 5: Monte Carlo sampling over microstates, each one conformer per residue or ligand, gives Boltzmann-weighted conformer probabilities and hence the protonation and tautomer populations of each system. Row 6: comparing the three systems reveals inhibitor-induced changes in residue charge state. Each of the three calculations is repeated three times independently (Trial01–Trial03) with a separate Monte Carlo seed, and the values reported throughout are means over those repeats.

The apoprotein calculation is obtained by removing the inhibitor after rotamer generation, so that the apo and holo calculations share an identical protein conformer set and differ only in the presence of the ligand. This construction is deliberate: it isolates the electrostatic consequence of removing the ligand at fixed geometry, so that every difference between the two calculations is attributable to the ligand and not to a conformational change. It is therefore not an independently sampled apo conformational ensemble, and we do not interpret the apo–holo differences as thermodynamic differences between independently equilibrated states.

The complete pipeline was repeated three times independently (Trial01–Trial03), each a full rerun of MCCE steps 1–4 from the same starting structures with its own Monte Carlo seed (MONTE_SEED 1001, 1002 and 1003). Each trial considers the three states (bound-kinase, apo-kinase and inhibitor in solution) for all 37 structures, for a total of 333 independent runs. We note that side-chain rotamer generation in step 2 does not reproduce between runs, and no structure returns the same total conformer count in all three trials, so a trial rather than a repeated Monte Carlo run counts as a complete replicate. We report the means and standard errors across all trials in Table 1. All values reported in Table 1 are means over the three trials. The per-trial values behind each of them, together with their means and standard errors, are tabulated in the Supporting Information (Table S4). A standard error of zero there means all three trials returned the same value, and indicates agreement rather than an unreported error.

MCCE calculations begin with an input PDB protein structure. The protein backbone is held fixed while side chains and ligands are assigned predefined choices for position and protonation states, where each choice is termed a conformer. Conformer selection thus defines the degrees of freedom in the calculation. One conformer for each residue or cofactor in the structure determines a microstate. Microstates are subjected to Metropolis–Hastings Monte Carlo sampling, and the resulting Boltzmann-weighted conformer probabilities yield the average protonation, tautomer, or positional state for each residue and ligand.

### MCCE Conformer Generation and Energy Calculations

The inhibitor heavy-atom positions are fixed, with protons placed differently in each conformer (Figure S1). The set of protonation and tautomer states available to the calculation is fixed before MCCE is run. It is the set that survives the Epik enumeration cutoff described above, a maximum state penalty of 10 kT, which is 5.9 kcal/mol at 298 K and is quoted as 6 kcal/mol throughout, relative to the lowest-energy state in solution at pH 7.4. Those states are assigned AM1-BCC charges and written into the MCCE ligand topology file, which is what defines the ionization and tautomer states MCCE may occupy.^26^ MCCE samples proton positions and heavy-atom rotamers for the states it is given and neither adds nor removes chemical states of the ligand; the prefiltering is an Epik step, not an MCCE one.

Two energies describe each inhibitor conformer: the Epik state penalty is the cost of forming that state in water, and the desolvation penalty is the cost of taking it out of water, measured against a calibrated solution reaction-field energy specific to the ε_prot_ = 4 used here.^29^ The solution ensemble is therefore a calibration point rather than a prediction, and the informative quantity is the shift away from it on binding. The full treatment, including the calibrated solution reference, the agreement of the sampled solution populations with the Boltzmann prediction over all 138 conformer entries, and how each term enters the microstate energy, is given in the Supporting Information.

Full rotamer sampling was applied to amino acid side chains. Side-chain conformers were generated by building rotamers without hydrogens, packing the protein repeatedly with them, and accepting rotamers on a fuzzy energy criterion based on an analytical electrostatic function rather than the Poisson–Boltzmann analysis used in the final Monte Carlo sampling.^26^ Protons were then added to the accepted rotamers to generate the protonation and tautomer states defined in the residue topology. This yields more neutral conformers than ionized ones, a single Asp rotamer spawning on average one ionized and four neutral, which would bias Monte Carlo sampling toward the neutral form if left uncorrected. MCCE removes it with an entropy correction evaluated within the sampling itself and iterated to convergence;^26^ the correction is computed for every calculation reported here. MCCE conformer generation is nondeterministic, so rather than relying on a single conformer set the entire pipeline was repeated three times independently, as described above, and every value reported here is a mean over those three trials.

Self-energies for each conformer and pairwise energies between all conformer pairs were calculated prior to Monte Carlo sampling.^26^ Self-energy terms are treated as constants for a given conformer, independent of conformer choices at other residues. The conformer self-energy includes torsion and internal van der Waals energies as well as the chemical energy for the protonation state at the pH of interest, Δ*m*_*i*_(pH – p*K*_a_), where *m*_*i*_ is the change in protonation number from the reference state. For the inhibitors these self-energy terms are replaced by the Epik state penalty of eqs 1 and 2, weighted as in eqs 4 and 5: the heavy atoms are fixed, so the torsion and internal van der Waals contributions are zero for every ligand conformer, and the ligand has no defined solution p*K*_a_. Additional self-energy terms include the self-electrostatic-desolvation penalty incurred upon the ligand moving from solution into the binding site and interacting with the fixed protein backbone. More detailed information can be found in the supplement ”Microstate energy used in Monte Carlo sampling”.

The differential binding free energy between two states *i* and *j* of the same inhibitor is obtained directly from their Boltzmann populations in the bound and solution ensembles

6 $$\Delta \Delta G\left(i\to j\right)=-RT\;\ln\frac{P{\left(j\right)}_{prot}/P{\left(i\right)}_{prot}}{P{\left(j\right)}_{soln}/P{\left(i\right)}_{soln}}$$

where the populations are those reported (Table S2). This is a relative quantity between two protonation or tautomer states of one ligand in one complex; it is not an absolute or alchemical relative binding free energy between different ligands. We note that the solution populations are governed by the Epik state penalties alone, so those penalties appear in both the numerator and the denominator and cancel: As such, ΔΔ*G* measures only the differential effect of the binding site on the two states, not the intrinsic cost of either. For the two +1 tautomers of imatinib in 3PYY the Epik penalties differ by 0.26 kcal/mol and predict a solution population ratio of 0.648, which the sampling reproduces as 0.646; the −3.5 kcal/mol differential reported for that complex is therefore a property of the protein environment.

Delphi was used to solve the Poisson–Boltzmann equation for electrostatic self- and pairwise interaction energies. The protein/ligand dielectric constant was set to 4 and the solvent to 80, with 150 mM implicit salt. PARSE charges and radii were used for electrostatic calculations,^37,38^ along with AMBER van der Waals and torsion parameters.^39^ Conformer probabilities were obtained through MC sampling.

### MCCE Microstate Analysis

In addition to conformer probabilities, MCCE saves all accepted microstates along with their energies and sampling counts.^40^ These microstates can be organized to characterize the protonation state distribution of the protein and to examine correlations between the protonation states of different residues (Figure 2). Each protonation microstate is defined by a unique combination of charge states across these residues. The ABL kinase domain contains 11 Asp, 28 Glu, 15 Tyr, 11 Arg, 19 Lys, and 6 His residues (Figure 2A). While most are fully ionized (>99% probability) at pH 7.4, several His and Tyr residues adopt mixed protonation states, generating 2448 unique protonation microstates in a total ensemble of 3,000,000 microstates with net charges ranging from −10 to −1 and a Boltzmann-weighted mean of −3.89 (Figure 2B). Despite this broad distribution, only 115,932 protonation/conformation microstates are needed to capture 50% of the equilibrium probability, and 209,236 microstates encompass 90%. Thus, while the protein cannot be described by a single protonation state, the ensemble is concentrated in a tractable region of protonation space. The Boltzmann-averaged distribution of inhibitor conformers is reported in Table S2.

**Figure 2:**
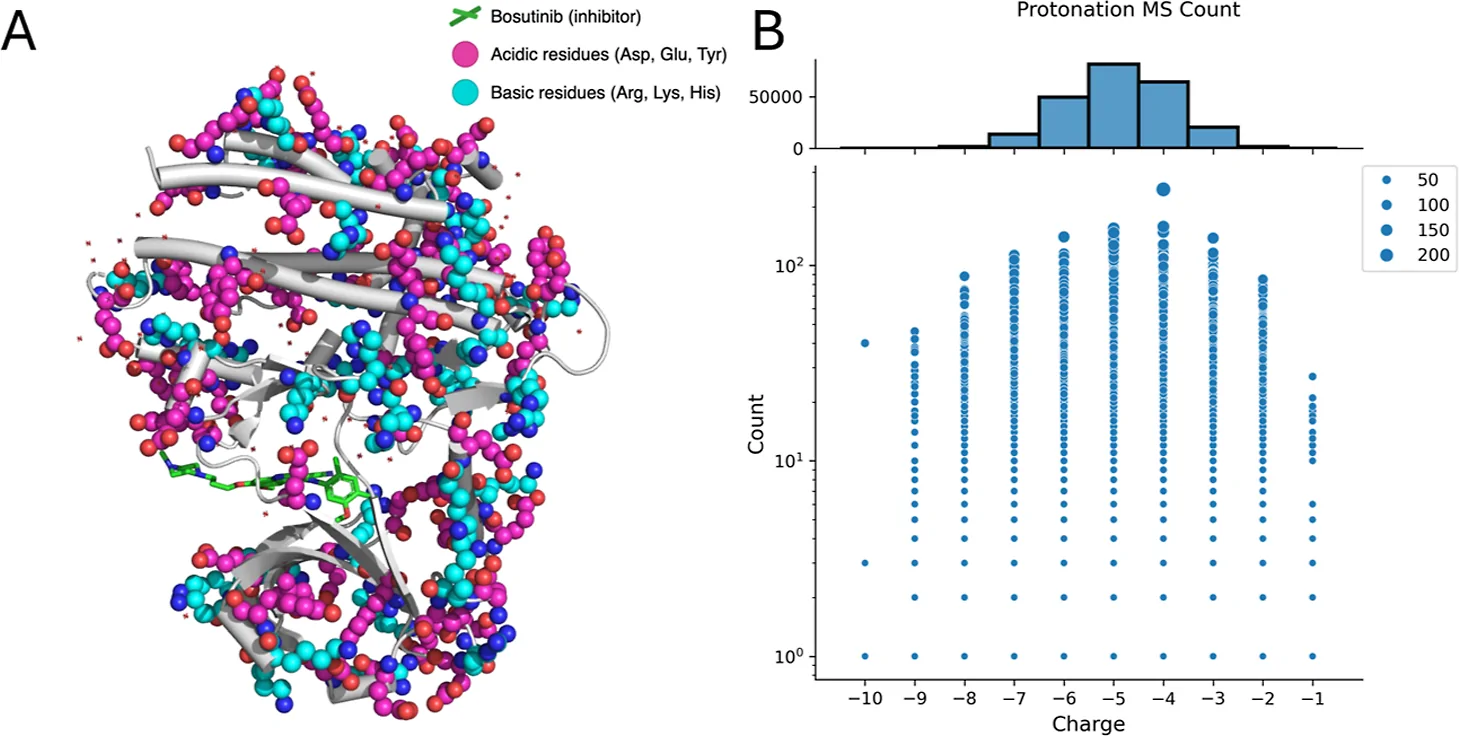
MCCE microstate distribution of bosutinib bound to the ABL tyrosine kinase domain. (A) Structure of the ABL tyrosine kinase domain bound to the inhibitor Bosutinib (PDB: 3UE4). The ionizable residues shown as spheres (acids: magenta; bases: cyan) and the inhibitor shown as green sticks. (B) MCCE microstate charge distribution at physiological pH (7.4). The upper histogram shows the total microstate count at each net charge; the lower panel displays sets of unique microstates, with point size proportional to their sampling count in the Monte Carlo ensemble.

## Results and Discussion

### Characterization of Ligands in Solution

The inhibitors studied here (Table 1) are all complex heterocycles with molecular weights of the order of ≈500 g/mol. Each has multiple protonation/tautomer states that are predicted to be within 6 kcal/mol of the lowest energy conformer at pH 7.4, and are thus thermally accessible. The number of included protonation/tautomer states is different for each inhibitor. Osimertinib has seven conformers, while three inhibitors have only two (Table S2). In solution at pH 7.4, the lowest energy state carries a charge of +1 for 11 inhibitors, and zero for six others.^41^ Crizotinib is the exception with a lowest energy state having a +2 charge. Only Lenvatinib and Sorafenib have negatively charged conformers within 6 kcal/mol of their lowest energy state, but these are rarely populated in MCCE simulations (probability <0.001). The Boltzmann-averaged inhibitor charge in solution ranges from 0 to 1.6 at pH 7.4 (Table S2).^42^ As expected, these solution values are a property of the ligand rather than of any particular complex.^29^ The inhibitor in solution is described by its Epik state penalties alone, and the MCCE solution calculation reproduces the Boltzmann distribution over those penalties, serving as a calibration point (Methods). The most informative quantity thus becomes the shift upon binding, which is the difference between the second and third values. Two consequences follow: the solution charge of an inhibitor is identical in every structure in which it appears, most clearly for the six gefitinib complexes that share a solution charge of 0.30, and it is identical in all three trials for all 38 sites, which is why Figure 3 has no horizontal spread. Everything that differs between complexes therefore arises in the bound state.

**Figure 3:**
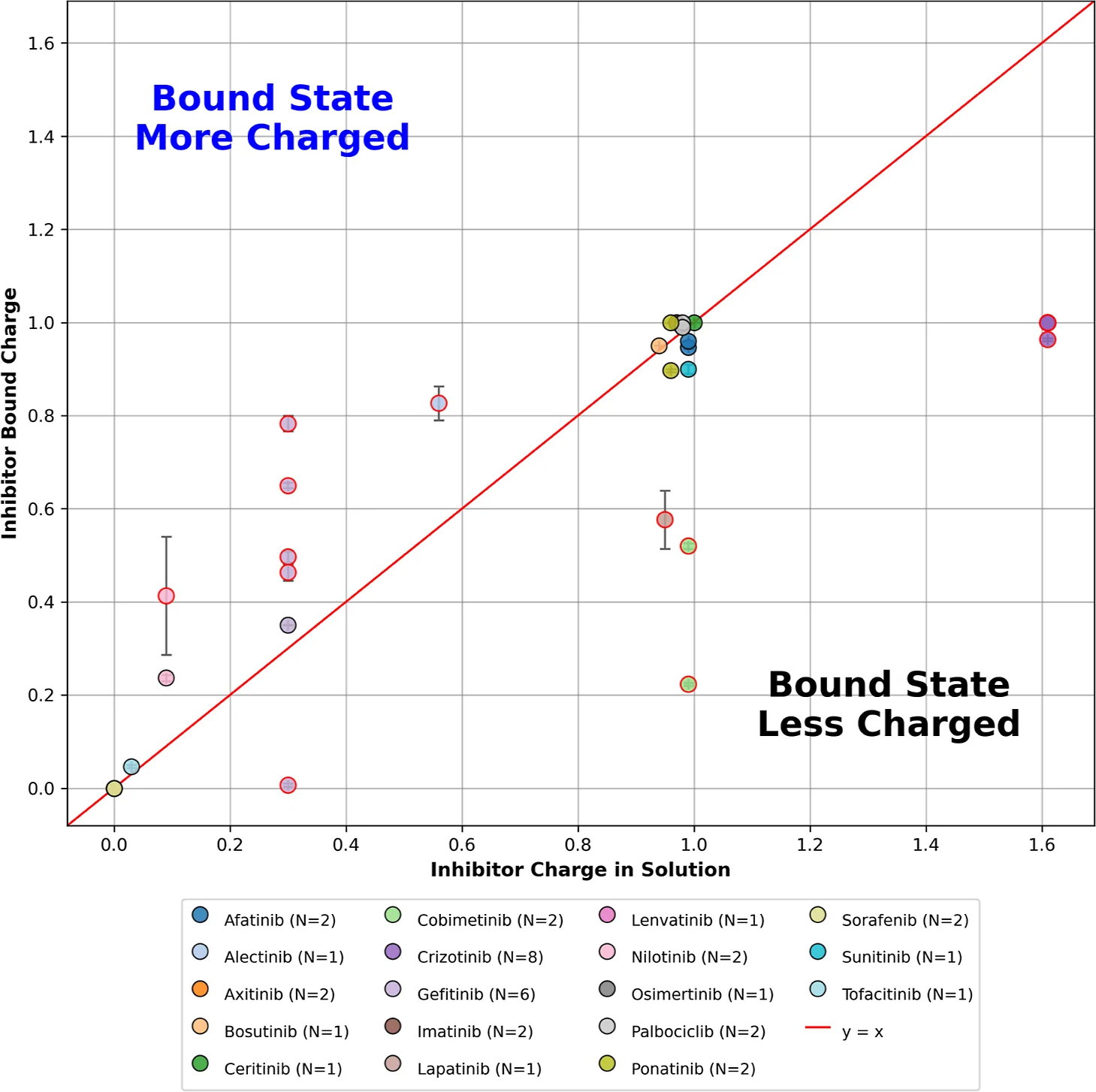
Inhibitor ensemble charge in the protein-bound state versus in solution, averaged over three independent repeats. Each point is one inhibitor–kinase pair; inhibitors are distinguished by color and *N* is the number of cocrystal structures analyzed for each. The 37 structures contribute 38 points because ponatinib occupies two sites in 3ZOS. Error bars are ±1 SEM; an absent bar means all three trials returned identical values. The red diagonal (*y* = *x*) marks equal bound and solution charge, so points above it gain charge on binding and points below lose it. Per-trial values are given in Table S4 and the individual values in Table 1.

Thermally accessible tautomers, which by definition have the same net charge, are considered simultaneously in the pipeline (Figure 1). Four of the inhibitors have two tautomers within 2.8 kcal/mol of the ground state, eight have tautomers only at higher energy, while six have no tautomers among the allowed conformers. We find that tautomers often have similar energy in solution, but can have significantly different protein affinities (Table S2).^43^

Our solution-state ligand charge-ensembles provide experimentally testable hypotheses. Since the solution populations are Boltzmann distributed over the Epik state penalties, the population ratio of two adjacent ionization states is equivalent to a p*K*_a_. We make this comparison (Table S6) against measured values for the 12 inhibitors found in the literature, using the equations given in the Supporting Information. It is worth noting that these are tests of a reference state rather than of the binding change itself; we are not determining p*K*_a_ directly since all calculations are run at a single pH. Consistency with measured values shows only that our solution ensembles and populations reflect the measured p*K*_a_ values. Our calculated values correlate well with previous measurements (Pearson *r* = 0.93) over a large range of 4.9 log units. Eight of the 11 deviations are positive, creating a mean signed deviation of +0.63 log units that indicates that the assigned solution states are systematically too basic. This is qualitatively consistent with the direction and magnitude of error expected for assigning amine-containing drugs.^42,44,45^

An offset of this size matters only where a site is poised near the sampled pH. For nine of the 12 inhibitors with prior measurements, the dominant solution charge is consistent with the measured values. Crizotinib is the one clear disagreement, a result of overestimating the aminopyridine by 2.0 log units, placing the dominant solution state at +2 against the measured +1; its piperidine p*K*_a_ is reproduced to within 0.1 log units. Alectinib and palbociclib are the remaining cautions, in both cases because the measured p*K*_a_ lies within 0.4 log units of 7.4, so the solution state is a near-even mixture whose majority component is not sharply defined. These three inhibitors are therefore the ones whose reported charge changes upon binding carry the largest uncertainty from the solution reference, and gefitinib, whose measured p*K*_a_ of 7.2 the calculation reproduces to within 0.2 log units, is poised for the same reason but with the reference correctly placed.

### Inhibitors Can Change Charge upon Kinase Binding

We consider the off-targeted kinase-domains of the larger protein (Table 1, Figure 3, Table S1, and Figure S1). Throughout Figure 3 and Table 1 the plotted value is the mean over the three independent repeats and the error bar is ±1 SEM over those repeats. Where no bar is visible the three trials returned the same value and the standard error is exactly zero, which indicates agreement between independent conformer sets rather than an unreported uncertainty; with *n* = 3 a 95% interval would be ±4.30 × SEM, so the bars are best read as a measure of spread.

Solution-state charges are identical in all three trials for every structure, so there is no spread in Figure 3. Two points, nilotinib in 3CS9 and lapatinib in 1XKK, show the largest trial-to-trial variation, so these carry the two largest bars and are best read alongside their per-trial ranges. Of the 38 ligand sites, 22 return the same bound charge in all three trials with a standard error of zero, while 16 vary across trials (Table S4). Consistently, across the 3668 per-residue apo-to-holo comparisons, 3179 (86.7%) are identical across trials, 488 (13.3%) are centered on a single value, and one, CYS775 in 1XKK, shows bimodal behavior (Table S3). The observed spread is explained by the population information collected at the single declared pH. A site divided between two states has occupancy variance *f*(1 – *f*), and ranking the 38 sites on that quantity reproduces the observed scatter, with a Spearman correlation of ρ = 0.84 against the trial-to-trial range; the derivation and the per-group means are given in the Supporting Information. A large spread therefore identifies a site whose population is genuinely divided across multiple states rather than a failed calculation, and such sites are better reported as a range than as a mean. For several, including ABL, ALK, EGFR, and VEGFR, there are examples with multiple inhibitors bound to the same proteins. Others, such as JAK3 and MET, have only a single example. We compute the ligand charge in solution and in the protein, as well as the charge of apo and holo (ligand-bound) protein forms (Figure 3 and Table 1). In solution, all inhibitor ensembles are predicted to have neutral or positive average charge. In MCCE, the VEGFR, MET and JAK3 apoproteins, as well as some EGFR structures, have a net positive charge, while the remaining structures are negatively charged.

Some inhibitors with low solution charge (charge <0.3) gain appreciable positive charge upon binding, while most inhibitors that are already substantially protonated in solution (charge ≥0.9) retain similar charge in the bound state. Notably, crizotinib (ensemble charge = 1.61 in solution) maintains its high charge (ensemble charge = 1.00) when bound. Solution charge and bound charge are correlated across the set (*r* = 0.83, ρ = 0.83), but that correlation does not carry the whole story, because the same inhibitor can take very different charges in different binding sites. Gefitinib is the clearest case: its solution charge is 0.30 in all six of its structures while the bound charge spans 0.01 to 0.78, which exhibits the diversity of binding-site electrostatic environments directly. Moving a charged ligand into a binding site penalizes the favorable electrostatic interactions between ligand and solvent.^46^ MCCE evaluates this explicitly: the desolvation penalty for a given inhibitor state is the Poisson–Boltzmann reaction field of that conformer in the site measured against its calibrated reaction-field energy in solution (Methods). The bound distribution is therefore set by a competition between three terms that the Monte Carlo sampling weighs together. The Epik state penalty is fixed and favors whichever state is cheapest in water. The desolvation penalty opposes burying charge and grows with the charge of the state. Electrostatic interactions in the binding site can compensate for this desolvation penalty, but they depend on which ionizable groups the pocket presents. A state that is costly in water can therefore dominate when bound, and we find that some proteins stabilize a ligand charge of +1 rather than zero (Figure 3). Which term prevails depends first on the energy difference between the neutral and charged forms. If the ligand p*K*_a_ is near the pH of interest, small changes in the environment can change the protonation state distribution of the ligand. If the energy levels are further apart, indicating that the ligand’s pH environment is further from the p*K*_a_, protein–ligand interactions must contribute more energy to shift protonation states. Furthermore, the protein binding site itself can dictate the protonation state: specific residues within the pocket may favor and stabilize either a positively or negatively charged ligand, effectively shifting its p*K*_a_ relative to the bulk solvent.

We highlight a few notable kinase-inhibitor complexes below.

#### Charge Changes are Specific to the Kinase and to the Site

VEGFR and MET apoproteins have positive calculated charges at pH 7.4, as do JAK3 and some EGFR structures (Table 1). Of the inhibitors bound to VEGFR, axitinib, lenvatinib and sorafenib are neutral both in solution and when bound, and sunitinib alone is charged in both. Axitinib shows only a modest tautomer shift, moving the probability that N15 rather than N14 is protonated by ≈15% (Figure S1, Table S2).

ALK prefers an inhibitor of charge +1 and reaches it from three different starting points. Alectinib has only two low energy conformers, one neutral and one +1, and sits near its p*K*_a_ at pH 7.4 in solution, 56% in the +1 state and 44% neutral, rising to 83% + 1 in the protein. Ceritinib begins 4 kcal/mol in favor of +1 and retains it on binding. Crizotinib is 62% + 2 and 38% + 1 in solution, yet bound to ALK the +1 conformer dominates and all others fall below 0.001. The same inhibitor behaves differently in a different site: bound to MET, crizotinib is a mixture of +1 and neutral conformers even though the neutral state lies 2.6 kcal/mol higher in energy, so MET does not stabilize crizotinib’s positive charge as ALK does.

EGFR and ABL show the same site specificity. The neutral form of lapatinib lies 1.74 kcal/mol above the +1 state, giving it only a 5% probability of being neutral in solution but 42% when bound; afatinib retains its charge of +1; gefitinib shifts to be somewhat more likely +1 than neutral when bound. ABL is bound to bosutinib, imatinib and nilotinib. Bosutinib and imatinib are predominantly +1 in solution and that form is stabilized in the protein, whereas nilotinib is predominantly neutral in solution, with an ensemble charge of 0.09, and becomes appreciably more cationic when bound, reaching 0.41 in 3CS9 and 0.23 in 5MO4.

#### Gefitinib Bound to EGFR is the Most Structure-Dependent Case in the Set

We analyze six cocrystal structures of EGFR–gefitinib and find that the gefitinib bound-state charge ranges from 0.01 in 3UG2 to 0.78 in 2ITO while the solution-state charge remains identical, 0.30, in all six. Since the calculation differs between them only in the input coordinates, the spread has to arise from the binding site itself.

In analyzing the structures of these complexes, we note that two acidic groups are positioned to stabilize the cationic form of gefitinib: Glu762 on the αC helix and Asp855, the aspartate of the DFG motif. In five of the six structures Asp855 lies 3.3–4.0 Å from the nearest inhibitor atom. Glu762 is engaged in four of them, at 3.2–3.4 Å, but sits 11.0 Å away in 4I22. In 3UG2, the structure that gives an essentially neutral ligand, both contacts are lost: Asp855 sits 6.8 Å away and Glu762 5.4 Å. The same disengagement is visible in the regulatory geometry, where the Lys745–Asp855 distance is 6.8 Å in 3UG2 against 2.8–4.1 Å in the other five. With neither of these two carboxylates positioned to compensate the desolvation penalty of burying a cation, the neutral tautomer dominates.

It is important to note that these are not general structural determinants driving charge changes. For example, Asp800 lies 3.1–4.4 Å from the inhibitor in all six structures, 3.4 Å in 3UG2 included, and so the mere proximity of a carboxylate does not set the bound charge. We hypothesize that 3UG2 differs through the loss of the Asp855 and Glu762 contacts specifically: across all six structures the distance to Asp855 correlates with the bound charge at *r* = −0.80, but that correlation is carried entirely by 3UG2, since excluding it gives *r* = +0.05 while the charges of the remaining five still range from 0.35 to 0.78. It is worth noting, however, that a simple Coulomb sum over the charged side chains within 12 Å of the protonation site does not account for the ordering either (*r* = +0.03), which is expected since it neglects both screening and the desolvation penalty that the full Poisson–Boltzmann treatment supplies. As such, while we can hypothesize about a structural explanation for the outlier, it is difficult to relate it to the range of values observed.

That residual spread is better understood as the poised-site behavior described above: gefitinib’s solution charge of 0.30 leaves its bound population near an even split rather than saturated in either form, the regime of maximum variance, and several of these structures show measurable trial-to-trial variation (Table S4). For a ligand in this regime the useful statement is not a single charge but that the bound population is close to an even split between the neutral and cationic forms, which is precisely the circumstance in which a pH-dependent binding measurement would be most informative.

### Change in Tautomer Distribution

Six of the 18 inhibitors have no tautomers calculated to be within 6 kcal/mol of the lowest energy configuration at pH 7.4.^47^ Four inhibitors (Axitinib, Bosutinib, Imatinib and Ponatinib) have two tautomers within 2.8 kcal/mol of the lowest energy state in solution. It is within this group that the most significant shift in conformer probabilities occurs on going from solution to the protein-bound state. Thus, in Imatinib-ABL or Ponatinib-DDR1, the lowest energy solution tautomer is not found when bound to the protein.^43^

The magnitude of these preferences can be quantified with eq 6. The clearest case is imatinib bound to ABL. Its two +1 tautomers STI + 1 and STI + 2 are nearly equally populated in solution (0.59 and 0.38), but when bound the equilibrium is almost completely resolved in favor of STI + 2, giving ΔΔ*G* = −3.5 ± 0.1 kcal/mol in 3PYY. In a second ABL structure, 2HYY, the resolution is more complete still: STI + 1 is not sampled at all in two of the three trials and reaches only 0.003 in the third, so the difference is bounded by the 3PYY value rather than determined by it. Ponatinib in DDR1 shows the same inversion more modestly (−1.1 ± 0.05 kcal/mol), as does bosutinib in ABL (−1.4 ± 0.01 kcal/mol), while axitinib bound to VEGFR is the clearest case in the opposite direction, the protein reinforcing rather than inverting the solution preference (+1.7 ± 0.05 kcal/mol in 4AGC and +1.0 ± 0.01 kcal/mol in 4AG8). Differentials of this size are comparable to the differences in binding affinity that structure-based design routinely tries to resolve, yet they arise entirely from which tautomer is assumed. All pairs for which the populations are large enough to determine a differential are tabulated in Table S5. While the specific binding pose of the ligand can influence the assessment of the net charge, these ligands typically adopt a single dominant pose, which dictates the overall net-charge distribution.

### A Small Fraction of Kinase Amino Acids Change Charge upon Inhibitor Binding

In these calculations the same protein coordinates are used for the holo- and apoprotein calculations, with the only difference being the presence of the ligand. Overall, we find that inhibitor binding does not change the vast majority of residue protonation states when comparing between apo and holo protein forms (Figure 4). Points falling on the identity line (red) indicate residues whose protonation state is unaffected by inhibitor binding (Figure 4). In turn, we find that the stability of the protein’s net charge upon ligand binding (Table 1) is not an artifact of compensating protonation changes at different sites. Rather than balancing out protonation changes across the protein, the net charge remains stable because most residues remain unchanged between apo and holo protein forms. Protonation shifts are limited to just a few residues.

**Figure 4:**
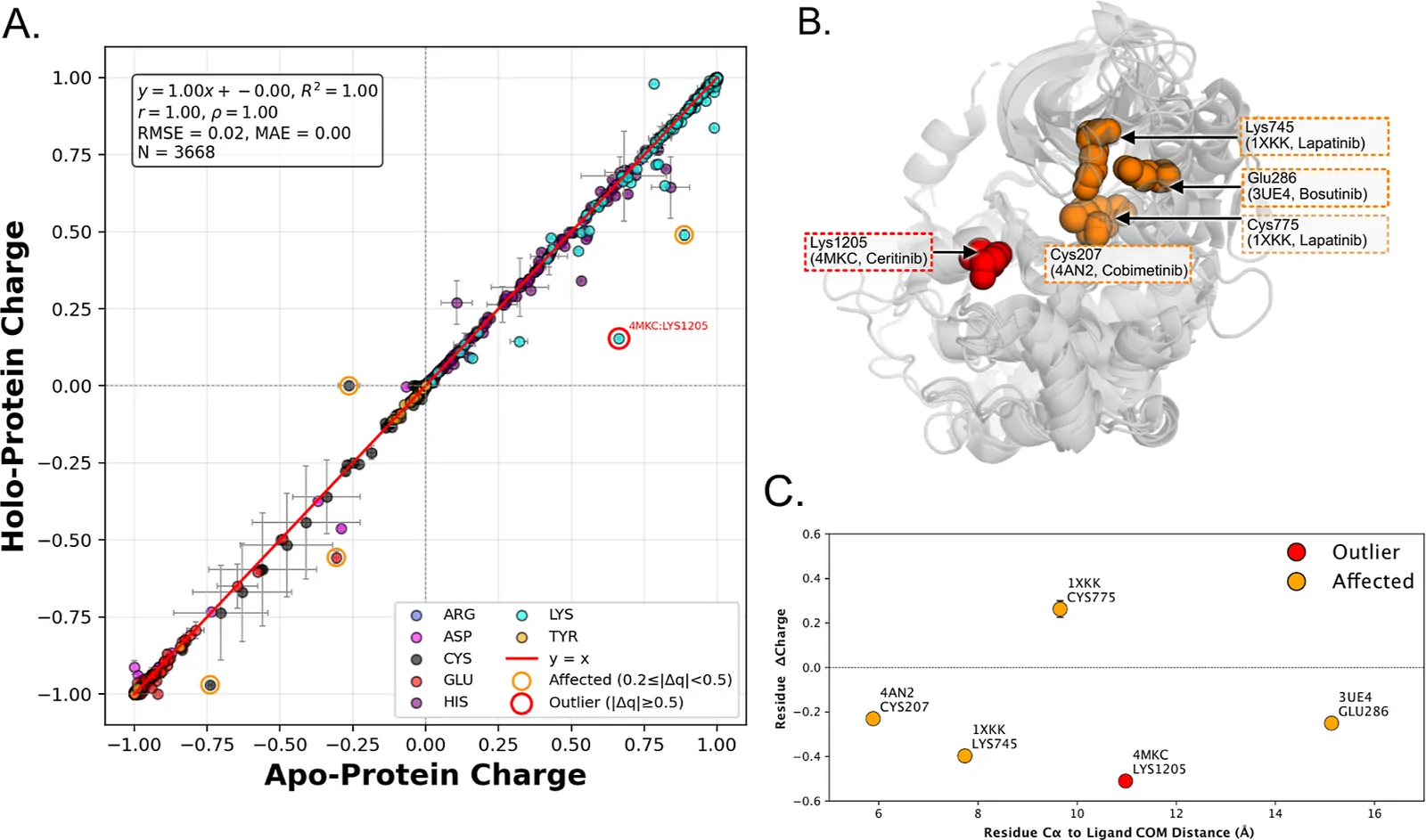
Effect of inhibitor binding on residue charge states across kinase–inhibitor complexes. (A) Calculated average charge of each titratable residue in the inhibitor-bound holoprotein versus apoprotein MCCE simulations at pH 7.4, aggregated across all kinase-inhibitor pairs (*N* = 3668 residue comparisons from 37 complexes). Points are colored by residue type and the red line denotes identity with no change between apo- and holo-protein. Each point is a mean over three independent repeats of the full pipeline and the bars are ±1 SEM over those repeats; where no bar is visible all three trials returned identical values. Residues whose charge shifts upon binding are highlighted in two tiers based on |Δ*q*| = |*q*_holo_ – *q*_apo_|: outlier residues (red open circles, |Δ*q*| ≥ 0.5) are labeled with PDB/residue, and affected residues (orange open circles, 0.2 ≤ |Δ*q*| < 0.5). (B) Structural overlay of all residues whose charge shifts upon binding are considered affected (orange, 0.2 ≤ |Δ*q*| < 0.5) or outlier (red, |Δ*q*| ≥ 0.5). All kinase structures with affected or outlier residue charge-changes are overlaid (gray cartoon), showing the charge-shifted residue from A in the respective color. For each charge-shifted residue, the residue identity, position in sequence, PDB ID and corresponding inhibitor are provided (box and arrow). (C) Distance dependence of the residue charge-change for affected (orange) and outlier (red) residues. Distance is measured from the inhibitor center of mass (”Ligand COM”) to the residue Cα atom. The corresponding PDB and residue index for each point are labeled.

Some amino acids, predominantly Lys and Cys residues, were observed to have significant shifts in protonation states upon inhibitor binding. We define “affected” residues as those exhibiting a change in charge (|Δ*q*|) between apo and holo states of 0.2 ≤ |Δ*q*| < 0.5, and “outliers” as those with |Δ*q*| ≥ 0.5 (Figure 4A). Accordingly, the majority of kinase complexes do not have a large change in total protein charge upon inhibitor binding by the same cutoffs, with the exception of six structures: 4MKC (ALK, −0.59), 1XKK (EGFR, −0.39), 4G5P (EGFR, −0.32), 3UE4 (ABL, −0.31), 4ZAU (EGFR, −0.31) and 2WGJ (MET, −0.24). These shifts are consistently negative, predicting that the kinase releases protons as it binds a cationic inhibitor, which is the expected direction. At the level of individual residues only one exceeds |Δ*q*| = 0.5: LYS1205 in the ALK kinase complex (PDB: 4MKC), which shifts by −0.510 and reproduces to an SEM of 0.000 across the three trials. The five residues that respond at all, their tier assignments and their trial-to-trial reproducibility are listed in Table S3.

These significant shifts are not restricted to the binding pocket. Measured from the inhibitor center of mass to the residue Cα, the five responding residues span 7.3–15.8 Å (Figure 4C): CYS207 in 4AN2 at 7.3 Å and LYS745 in 1XKK at 7.7 Å sit against the pocket, whereas LYS1205 in 4MKC lies at 10.8 Å and GLU286 in 3UE4 at 15.8 Å. While the proximity of most shifted residues suggests that dynamic titration of the immediate binding-site shell captures the majority of the protonation response, our results demonstrate that inhibitor binding can also exert far-reaching effects on the net charge of distant residues.

### Charge Changes upon Binding are Largely Insensitive to Kinase DFG Conformation

Kinases exist in a variety of different configuration states, as captured and labeled in the PDB.^48^ The kinase-inhibitor pairings studied here encompass a range of structural configurations. It remains unclear if charge changes in protein or drug upon binding differ given a particular conformational state of a kinase.

Here, we split according to their DFG-state label, considering DFG-in states (some of which are active) and DFG-out states. While more DFG-in state kinases were used in this study, this is representative of the structures available in the PDB. However, 11 DFG-out structures were included. It is important to note that the same kinase can be present in our data set as two individual, conformationally distinct structures. For example, ABL and EGFR structures are found in both DFG-in and DFG-out states. While some kinase inhibitors are considered more conformationally adaptable than others, most inhibitor conformational selectivity ensures that kinase-inhibitors tend to be crystallized in a single DFG state.

Consistent with the identification of affected and outlier charge changes above, we observe that the kinases do not change charge magnitude upon drug binding, whereas the kinase inhibitors do. We can visualize this conformational dependence by plotting empirical cumulative distribution functions (ECDFs) of the data along this split, observing the spread with which net-charge changes in either inhibitor or kinase are observed upon binding (Figure 5). DFG-out conformations show very little change in inhibitor charge upon drug-binding at all, with the vast majority of charge change near zero.

**Figure 5:**
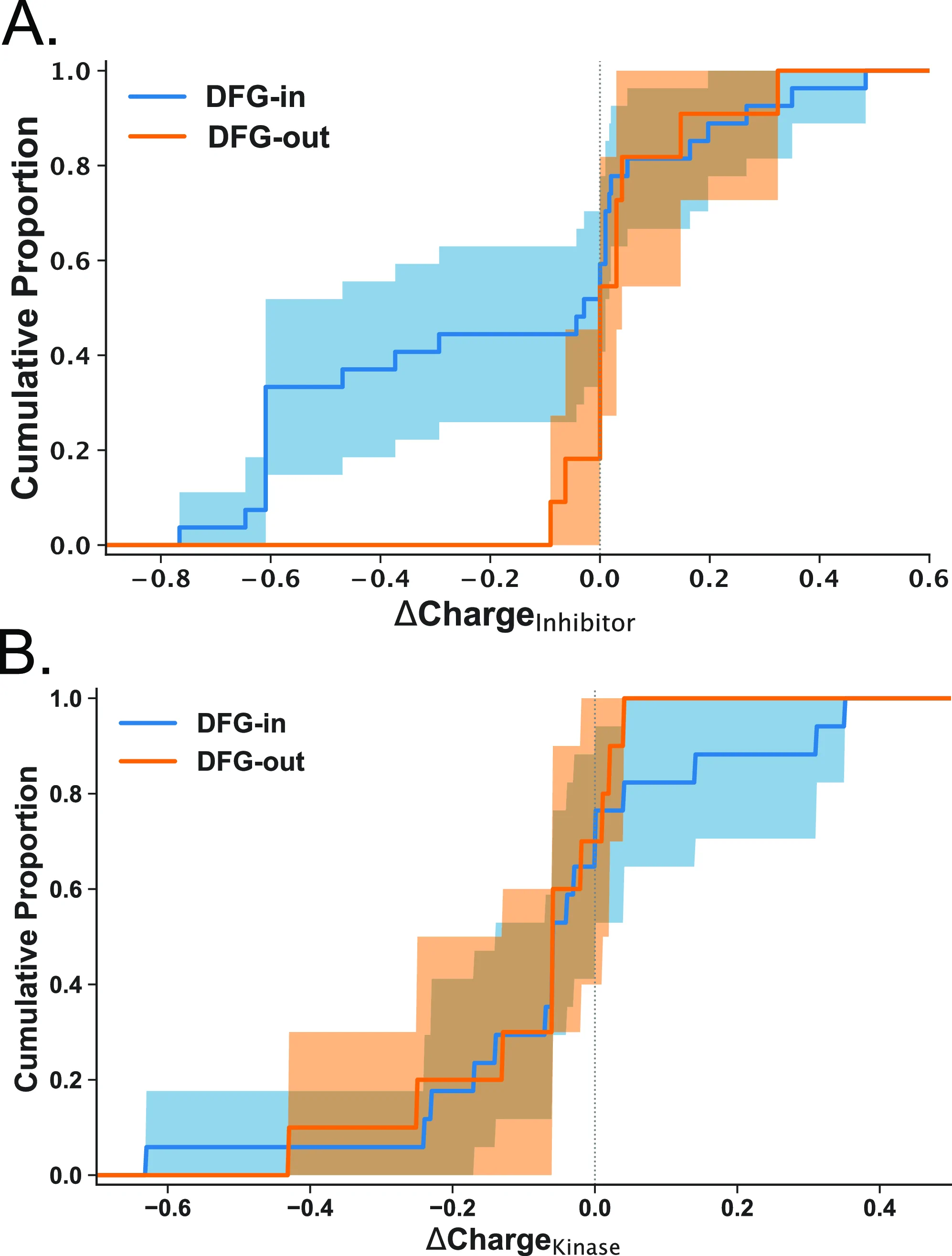
Net-charge changes of inhibitor and kinase on binding, split by DFG conformation. (A) Empirical cumulative distribution function (ECDF) of the change in inhibitor net charge on binding, the difference between the bound and solution values of Table 1. (B) ECDF of the change in kinase net charge on binding, the difference between the holo and apo protein values of Table 1. In both panels the structures are split by the DFG label of the bound complex, DFG-in (blue) or DFG-out (orange); charge changes of either sign are included, the vertical dashed line marks no net-charge change, and the shaded region is the 95% confidence interval about the ECDF (solid line). Both panels use values averaged over the three independent repeats, and the per-trial values are given in Table S4.

We observe that some DFG-in selective inhibitors experience a net-charge change upon binding, but the DFG-in kinase structures do not experience a significant change upon binding to an inhibitor. However, this analysis of the broad ensemble may interpolate over any subtle changes in microstate net-charge preferences, as was highlighted when identifying outliers (Figure 4). We note that this broad range of charge changes in DFG-in conformational states may be reflective of the increased structural diversity within DFG-in states. Dunbrack showed that there are multiple possible DFG-in states, only some of which are active.^48^

In summary, neither kinases nor ligands display a clear conformational dependence for charge changes upon drug binding. Some DFG-in inhibitors do become more charged on binding, but only a subset of them do, so the conformational state is not predictive of the degree of charge change in either partner. A practitioner should therefore treat the choice of target conformation and the net-charge change on binding as independent considerations, and read net-charge changes alongside other measurements rather than from the conformational label alone. We find that none of the DFG loop residues (Asp-Phe-Gly) themselves show significant protonation state changes upon inhibitor binding in any of the structures studied, consistent with the overall insensitivity of net charge to DFG conformation.

## Scope and Limitations of the Model

The calculations presented here make four simplifying choices, each of which bounds what the results can be taken to show.

### Fixed Backbone and Ligand Position

The protein backbone and the ligand heavy atoms are held at their crystallographic coordinates while side-chain rotamers, ligand protonation and tautomer states, and all polar proton orientations are sampled. Since the reaction-field contribution to the desolvation penalty is therefore evaluated for a single pose rather than averaged over a relaxed ensemble, the penalty will be overestimated for a charged state whose true bound geometry would relax to expose the ionizable group to solvent, and underestimated where relaxation would bury it further. Two things bound the size of this error. First, it enters only one of the terms that set the bound distribution. The Epik state penalty is a property of the ligand in water and is unchanged by the pose, so rigidity cannot bias the intrinsic cost of a protonation or tautomer state; it acts on the desolvation penalty and on the interactions with the protein, not on the state penalty itself. Second, the geometry is not rigid at the level that matters most for protonation: the heavy atoms are fixed, but every polar proton position and every ligand protonation and tautomer state is sampled explicitly, so the ionizable groups themselves are free to reorganize. The inhibitors considered here are also rigid, highly conjugated heterocycles that adopt a single dominant pose in each cocrystal structure. We therefore expect this to be a second-order effect relative to the charge-state differences we report, but it is not zero.

### The Apo State is the Holo Structure with the Ligand Removed

As described in the Methods, the apo calculation shares the holo conformer set so that the only difference between them is the presence of the ligand. This is the correct construction for the question asked here, which is how the protonation equilibrium responds to the ligand; it is deliberately not a model of the unliganded protein as it exists in solution. Relaxation of the apo protein would change side-chain positions, and any protonation-state change observed in such a calculation would confound the electrostatic effect of the ligand with the electrostatic effect of the conformational change, so the per-residue comparison in Figure 4 would no longer be attributable to binding. Separating those two contributions requires an independently equilibrated apo ensemble, and ideally apo cocrystal structures of the same kinase domains, which is a distinct study and outside the scope of this work. We note it as a natural extension: comparing a relaxed apo ensemble against the fixed-geometry result reported here would quantify how much of the protonation response survives conformational relaxation.

### Continuum Solvent in Place of Explicit Waters

Crystallographic waters were removed and the solvent represented as a dielectric continuum. Continuum solvation is a well-established component of MCCE and has been successfully used to describe solvent screening, reaction-field energies, desolvation penalties, and electrostatic contributions to protonation and binding equilibria. Previous MCCE studies have demonstrated that this treatment, combined with explicit sampling of protein and ligand conformers, can reproduce experimental protein p*K*_a_ values and energetics without requiring an explicit representation of bulk water.^25–27^ More recent applications have likewise used MCCE continuum electrostatics to calculate relative ligand and pigment binding affinities, including chlorophyll selectivity in LHCII and far-red-light photosystem II.^30,31^

The continuum representation does, however, have an important limitation: it cannot reproduce a specific, orientationally ordered water molecule that donates or accepts a defined hydrogen bond. Where a conserved pocket water bridges an inhibitor to the hinge or to a catalytic residue, omitting it may underestimate the stabilization of the protonation state that would have formed that hydrogen bond. It may also alter the local desolvation contribution because continuum solvent can treat an accessible cavity as solvent-filled without explicitly accounting for the thermodynamics and orientation of an individual confined water. The direction and magnitude of the resulting error therefore depend on the location of the water and on which inhibitor protonation or tautomeric state it preferentially stabilizes.

We note that MCCE can retain explicit waters as rotatable and protonatable conformers when their positions are sufficiently well-defined. Due to the variance in resolution across the 37 crystallographic structures, we elected to omit all waters for consistency. We note that water occupancy, resolution, and assignment vary substantially among these structures, and selectively retaining crystallographic waters only where they happen to be resolved would introduce further structure-dependent biases into the comparison. The present protocol consequently treats bulk solvent consistently through continuum electrostatics while accepting that specific water-mediated interactions are not explicitly represented.

### Choice of Protein Dielectric Constant

All MCCE calculations in this study use ε_prot_ = 4 with ε_solv_ = 80. In continuum electrostatics the protein dielectric constant is an effective parameter standing in for dielectric response not treated explicitly, so its appropriate value is coupled to how much conformational relaxation the calculation already includes: in a rigid protein representation a larger value can compensate for reorganization that is otherwise absent. MCCE instead combines continuum electrostatics with explicit sampling of side-chain rotamers, hydroxyl and proton positions, and alternative protonation states, allowing part of the local response to a change in charge to occur directly through the conformational ensemble rather than being represented entirely through an increased dielectric constant. The coupling is also structural rather than merely conceptual: the desolvation penalty is the difference between a conformer’s reaction field in the protein and its reaction-field energy in solution, and that solution reference is itself evaluated at the protein dielectric constant and stored per ionization state in the topology.^26^ Changing ε_prot_ therefore requires the matching solution reference, and for the inhibitors the calibrated reference as well; it is not a single parameter that can be varied on its own.

The choice of ε_prot_ = 4 follows the established MCCE parametrization and was explicitly examined in the MCCE2 p*K*_a_ benchmark.^26^ In that work, calculations using ε_prot_ = 4 and 8 were compared, and increasing the dielectric constant provided little improvement in the overall agreement with experimental p*K*_a_ values. This relative insensitivity was attributed to the explicit conformational degrees of freedom sampled by MCCE, which capture a substantial component of the local protein relaxation that a higher effective dielectric would otherwise represent. Importantly, the two dielectric constants were not completely equivalent. Increasing ε_prot_ from 4 to 8 weakens both reaction-field penalties and electrostatic interactions with the surrounding protein, systematically moving calculated p*K*_a_ values toward their solution values. Consequently, the larger experimentally observed p*K*_a_ shifts were reproduced somewhat better with ε_prot_ = 4: the slope relating calculated and experimental p*K*_a_ shifts was approximately 0.7 at ε_prot_ = 4, compared with 0.6 at ε_prot_ = 8.^26^

Thus ε_prot_ = 4 is not a claim that the protein interior behaves as a homogeneous material of dielectric four; it is the effective value benchmarked for MCCE’s sampling regime. Individual sites with protonation equilibria close to the sampled pH do remain sensitive to electrostatic screening, so the populations and fractional charges of such poised sites identify environments near a protonation transition rather than exact occupancies.

### Comparison with Measured p*K*_a_ Values

While solution states assigned to inhibitors are testable, it is much more challenging to test bound-state p*K*_a_ values in as systematic a manner. The quantity that would be required, the p*K*_a_ of an inhibitor site inside a kinase active site, has not been measured for any of the complexes studied here, so the charge and tautomer changes reported in this work are predictions. We note that one route to testable measurements for these predictions is to use pH-dependent binding affinity assays with a pH-insensitive reporting technique such as fluorescence, calorimetry or surface plasmon resonance.^13^ Such a data set of measurements does not exist in the literature, based upon our searching. While other techniques such as NMR have also been used to measure p*K*_a_ values, it remains challenging to measure changes in protein and ligand p*K*_a_ values and charge ensembles upon binding.

## Conclusions

Protonation and tautomeric state changes are important but often neglected contributors to protein–ligand binding in most structure-based modeling workflows.^47^ In general, including these degrees of freedom increases the complexity and cost of the calculations. Here, MCCE calculations are carried out on 37 crystallized structures of 9 different kinase domains bound to 18 different FDA approved inhibitors.

We find that the ligand protonation state does shift substantially upon binding and cannot be assumed from solution-state calculations alone. One outcome is that ligands often do not become less charged when bound. A general understanding of the factors that govern molecular binding is that charged ligands lose favorable solvation free energy when they are removed from water. This helps stabilize more neutral forms of the ligand, and the MCCE calculation is used to account for this energy change. However, interactions with other charges in the protein binding site can compensate. Here, it is found that many of the ligands retain a charge of +1 and others become more charged on binding. Thus, the appropriate net charge for the ligand is not always the one with lowest energy in water or a neutral form.

In contrast, we find that most proteins change protonation by a small amount even when a charged ligand is bound. There are multiple heuristics to explain this result. One is that although binding a charged ligand shifts the proton affinity of nearby residues, that shift need not produce a measurable change in protonation. At pH 7.4, near physiological pH, the pKas of Asp, Glu, Arg and Lys are often well below or above the pH, so these residues can have a weaker proton affinity without measurable proton release.^49^ The protein samples an ensemble of protonation microstates, but most individual residue populations change little upon ligand binding (Figure 4), consistent with the limited net-charge changes in Table 1. Similar net charges do not establish that different protonation microstates contribute equally to binding energetics.

In general only a small number of residues near the binding site show significant protonation changes upon inhibitor binding (Figure 4), dynamic titration of a limited binding-site shell may be sufficient to capture most of the protonation response without the cost of titrating the entire protein. Additionally, the DFG loop conformation does not significantly influence protonation state changes, and the DFG loop residues themselves do not undergo measurable charge shifts upon inhibitor binding.

Our results remain consistent and robust across three independent replicates, showing that these conclusions are not artifacts of a single calculation. Although side-chain rotamer generation is stochastic and no structure reproduces its conformer count across all three trials, the large majority of reported quantities are identical in every trial. Ligand trial-to-trial variation is associated with mixed bound-state populations at the pH studied. Across the 38 sites, *f*(1 – *f*), the variance of a binary state indicator, correlates with the trial-to-trial range (Spearman ρ = 0.84). This association does not by itself establish convergence or exclude sampling limitations. Sites with mixed populations and substantial trial-to-trial variation are useful candidates for pH-dependent binding measurements.

These conclusions are drawn at fixed backbone and ligand geometry, with the apo state modeled as the holo structure with the ligand removed. They therefore describe how the protonation equilibrium responds to the ligand itself, and not how it would respond to the conformational relaxation that accompanies binding in solution; separating those two contributions is a natural extension of this work.

Together, these results indicate that protonation and tautomeric state assignment should be treated as a binding-site-specific property of each kinase–inhibitor complex rather than assumed from solution-state ligand energetics, global kinase conformation, or a single fixed protonation-state model.

## Supplementary Material



## Notes

Supporting Information derivation of the electrostatic free energy in MCCE; the microstate energy expression used in Monte Carlo sampling; treatment of inhibitor conformer energies, covering the Epik state penalties, the calibrated reaction-field energies, and the reproduction of the solution ensemble; 2D chemical structures and sampled protonation and tautomer states of all 18 inhibitors, with Epik energies and ensemble charges (Figure S1); the kinase structures used, with residue ranges, chains and ionizable-residue counts (Table S1); conformer charge states, Epik energies and MCCE calculated solution and bound occupancies for every inhibitor (Table S2); residues whose protonation changes on inhibitor binding, with tier assignments and trial-to-trial reproducibility (Table S3); per-trial values, means and standard errors for every quantity in Table 1 (Table S4); differential binding free energies between tautomers of the same inhibitor (Table S5); solution protonation states of the inhibitors compared with measured p*K*_a_ values (Table S6) (PDF)

## Notes

The source code and data supporting the findings of this study are available in the following repositories: software: the MCCE4-Alpha software, which includes core topology files used for electrostatic calculations, can be found at https://github.com/GunnerLab/MCCE4-Alpha. Inhibitor parameters: the Epik-calculated inhibitor charges and related scripts are available at https://github.com/choderalab/mcce-charges/tree/master/epik_inhibitors. Project data: all kinase-specific data, simulation input/output files, and postprocessing scripts are hosted at https://github.com/granepura/Kinases_MCCE. Structural data: The specific PDB accessions from which the starting structures were derived are detailed in Table 1.
