# Supplementary material for: Kinase Inhibitors can Change Protonation or Tautomeric State upon Binding
Source: J Chem Inf Model. 2026 Oct 16;66(21):14159–73. doi: 10.1021/acs.jcim.6c02792 (PMC13753512; doi:10.1021/acs.jcim.6c02792)
Supplement: Supplementary file 1 [file ci6c02792_si_001.pdf]

Supporting Information
Kinase inhibitors can change protonation or tautomeric state upon binding

Gehan A. Ranepura,[†,‡] Saqibul I. Chowdhury,[‡] Elizabeth A. Rosenzweig,[‡] Ariën S. Rustenburg,[¶,§] Raquel López-Ríos de Castro,[‖] Junjun Mao,[⊥] John D. Chodera,[¶] Sukrit Singh,[*,¶] and M. R. Gunner[*,†,‡]

†*Ph.D. Program in Physics, The Graduate Center, City University of New York, New York, NY 10016, USA*

‡*Department of Physics, City College of New York, New York, NY 10031, USA*

¶*Computational and Systems Biology Program, Sloan Kettering Institute, Memorial Sloan Kettering Cancer Center, New York, NY 10065, USA*

§*Graduate Program in Physiology, Biophysics, and Systems Biology, Weill Cornell Medical College, New York, New York 10065, USA*

‖*Department of Physics, Freie Universität Berlin, Arnimallee 14, Berlin, 14195, Germany*

⊥*Benjamin Levich Institute for Physico-Chemical Hydrodynamics, City College of New York, New York, NY 10031*

E-mail: sukrit.singh@choderalab.org; mgunner@ccny.cuny.edu

Detail of Methods Used

Electrostatic free energy in MCCE

DelPhi[1–4] provides numerical solutions to the Poisson Boltzmann equation to determine the electrostatic interactions. The dielectric constant of four is used for all atoms. A dielectric constant of 80 is used for the external solvent and for internal cavities with a radius >1.4 Å. The salt concentration is 150 mM with a Stern layer of 2 Å. Thus, the dielectric response in MCCE includes both the implicit dielectric constant used in DelPhi and explicit response allowed by side chain and ligand conformers which sample of multiple dipole orientation. AMBER parameters[5,6] were used for van der Waals and torsion energies. The van der Waals scaling factors were 1.0, and the torsion-energy scaling factor was 0.125.

Microstate energy used in Monte Carlo sampling

The pre-made conformers of all residues, cofactors (inhibitors) are subjected to Monte Carlo (MC) sampling to generate a Boltzmann distribution of conformers. A microstate consists of one conformer per residue or cofactor. Metropolis-Hastings Sampling is performed to determine its acceptance.[7] The energy ΔH^x of microstate x is:

$$\begin{aligned}\Delta H^x = \sum_{i=1}^{M} \delta_{x,i} \Bigg\{ & \Big[2.3\, m_i k_B T \,(\mathrm{pH} - \mathrm{p}Ka_{,\mathrm{sol},i}) + n_i F\,(E_h - E_{m,\mathrm{sol},i})\Big] \\ & + \Big[\Delta\Delta G_{\mathrm{solv},i} + \Delta G^{\mathrm{CE}}_{\mathrm{bkbn},i} + \Delta G^{\mathrm{LJ}}_{\mathrm{bkbn},i} + \Delta G_{\mathrm{torsion},i} + \Delta\Delta G_{\mathrm{SAS},i}\Big] \\ & + \sum_{j=i+1}^{M} \delta_{x,i} \left[\Delta G^{\mathrm{CE}}_{ij} + \Delta G^{\mathrm{LJ}}_{ij}\right] \Bigg\} \end{aligned} \tag{S1}$$

where, M is the total number of conformers. The first line in Equation S1 describes the electrochemical properties of each conformer, where $\delta_{x,i}$ is 1 if conformer i is present in the microstate or 0 otherwise. The ability of the group to participate in acid/base reactions is

described for protons in the first term and redox reactions in the second term. m_i is 1 for protonated bases, −1 for deprotonated acids, and 0 for all neutral conformers. $pKa_{sol,i}$ is the reference pKa of this residue type in solution, and $Em_{sol,i}$ is the reference electrochemical midpoint potential of this redox active group.

k_B is the Boltzmann constant, T is the temperature, set to 298.15 K in these calculations, F is the Faraday constant, and n_i is the change in the number of electrons on a conformer type that is taking part in a redox titration. pH and Eh are the relevant solution parameters describing the chemical potential of protons or electrons that will come to equilibrium with the protein. The second line of Equation S1 describes the conformer self-energies of the microstate, which are independent of the other conformer choices of other residues and cofactors. These energies are the loss of the conformer's solvation energy as it moves from the referenced solvent to its position in the protein ($\Delta G_{solv,i}$), continuum electrostatic (CE) and Lennard-Jones (LJ) van der Waals interactions with the fixed backbone amides, torsion energy and the favorable LJ interactions with the exposed surface of the cofactor/residual sidechain with the implicit solvent, respectively.[8] The third line gives the CE and LJ pairwise interactions between the conformer and all other conformers selected in the microstate.

Inhibitor conformer energies: Epik state penalties and the calibrated reaction field

Two quantities determine how an inhibitor conformer is treated in Equation S1, and they enter at different places.

The first is the Epik total state penalty ΔG_{total}, the free energy cost of forming that protonation or tautomer state in water. MCCE provides a correction term in the microstate enthalpy of Equation S1 for conformer energies that the standard terms do not capture, and for the inhibitors that term carries ΔG_{total}, one value per state. It is the quantity that stands in for the solution acid/base term available to an amino acid of known pK_a, and it is identical in the solution and the protein-bound calculations because it describes the ligand

in water in both cases.

The second is $\Delta\Delta G_{\mathrm{solv},i}$, the change in reaction field energy on moving the conformer from water into its position in the protein. MCCE evaluates this as the difference between the Poisson–Boltzmann reaction field of the conformer in place and a standard reaction-field energy in solution held, per ionization state, in the residue or ligand topology file.[7] That solution reference has to be evaluated at the same protein dielectric constant used in the calculation, so the topology stores a separate value for each; the $\varepsilon_{\mathrm{prot}} = 4$ entry is the one used here. For the inhibitors these solution reaction-field energies were calibrated per conformer. That calibration was carried out before the solvation energies were assigned, with the Epik penalty in the correction term as the only quantity describing a conformer, and the resulting values were written into each inhibitor's topology file.

The two are not a double count. As such, the Epik penalty is the price of forming the state in water, while the desolvation term is the price of taking that state out of water and into the binding site.

This construction makes the isolated inhibitor a direct test of the treatment. In the solution calculation the reaction field is the reference state, so $\Delta\Delta G_{\mathrm{solv},i}$ is zero, as are the torsion, Lennard-Jones and backbone terms. The Epik penalty is then the only quantity distinguishing one conformer from another, and Monte Carlo sampling must return the Boltzmann distribution over those penalties,

$$P(i) = \frac{e^{-\Delta G_i/RT}}{\sum_j e^{-\Delta G_j/RT}}, \tag{S2}$$

with $RT = 0.5925$ kcal/mol at 298 K. Equation S2 is the same Boltzmann relation used in the Methods of the main text, written with the sum over conformers made explicit. It does. Across all 138 conformer entries in Table S2, which comprise 63 distinct conformers of the 18 inhibitors listed once per structure, the sampled solution occupancies match the Boltzmann prediction to a mean absolute difference of 0.001 and a maximum of 0.015, with 99% agreeing

to within 0.01. The single largest deviation is ponatinib, whose solution calculation is the only one containing two copies of the ligand, the two 3ZOS sites, so that the copies interact weakly with one another and their populations are not governed by the state penalties alone. This is the agreement between the charge and $P(i)_{\mathrm{soln}}$ ensemble values reported in Table S2. The solution ensemble is therefore a calibration point rather than an independent prediction, and the quantity that carries information is the shift away from it when the ligand is placed in the binding site.

Effective solution pK_{a} from the Epik state penalties

Table S6 compares the solution states assigned here with literature reference pK_{a} values. Because no titration is carried out in this work, this subsection sets out exactly how a pK_{a} is obtained from quantities the calculation does produce.

Every calculation reported here is run at a single pH, 7.4, so no pK_{a} is fitted. What the solution calculation provides is the Epik total state penalty $\Delta G_{total,i}$ of each inhibitor conformer i, tabulated for all 138 conformer entries in Table S2. In solution these penalties are the only quantity distinguishing conformers, so the conformer populations follow from Equation S2. Grouping those conformers by net charge q gives the population of each ionization state,

$$P(q) = \sum_{i\,:\,q_i=q} P(i) = \frac{\sum_{i\,:\,q_i=q} e^{-\Delta G_{total,i}/RT}}{\sum_j e^{-\Delta G_{total,j}/RT}}, \tag{S3}$$

where the denominator runs over every conformer of that inhibitor. Tautomers of the same net charge are therefore combined, so what follows is a macroscopic constant for the charge transition and not a microscopic constant for one atom.

For two states differing by a single proton, the Henderson–Hasselbalch relation rearranges to give the pK_{a} directly from that ratio,

$$\mathrm{p}K_{\mathrm{a}} = \mathrm{pH} + \log_{10} \frac{P(q{+}1)}{P(q)}, \tag{S4}$$

with pH = 7.4. Equations S3 and S4 can be combined into a form that uses the penalties alone. Defining the free energy of a charge state as $G(q) = -RT \ln \sum_{i:q_i=q} e^{-\Delta G_{total,i}/RT}$ and $\Delta G_{q\to q+1} = G(q+1) - G(q)$,

$$\mathrm{p}K_\mathrm{a} = \mathrm{pH} - \frac{\Delta G_{q\to q+1}}{2.303\, RT}, \tag{S5}$$

with $2.303\, RT = 1.364$ kcal/mol at 298 K. Equation S5 is how the pK_a(calc) column of Table S6 was evaluated, working from the penalties rather than from the rounded populations printed in Table S2.

Gefitinib illustrates the whole chain. Its five conformers carry Epik penalties of 0.212 kcal/mol (neutral), 0.717, 3.728 and 4.395 kcal/mol (+1), and 4.412 kcal/mol (+2) (Table S2). Equation S2 converts these to conformer populations, and Equation S3 groups them to $P(0) = 0.699$ and $P(+1) = 0.301$, the +2 state being unpopulated; Table S2 prints these as 0.700 and, summing its three +1 conformers, 0.300. The corresponding free energy difference is $\Delta G_{0\to +1} = +0.500$ kcal/mol, so Equation S5 gives $7.4 - 0.500/1.364 = 7.03$, reported as 7.03 in Table S6 against a measured 7.2.

Working from the penalties matters for the inhibitors whose minor state is barely populated, because there Equation S4 evaluated on populations rounded to three decimal places is no longer accurate. Axitinib is the clearest case: its +1 state is printed as 0.003 in Table S2, and using that rounded value would give 4.9 rather than the 4.6 that the penalties imply, an error of 0.3 log units. Ceritinib, sorafenib and ponatinib shift by 0.05 to 0.09 log units for the same reason. A reader recomputing Table S6 from the printed populations alone should therefore expect small discrepancies wherever P is close to 0 or 1.

The comparison requires the relevant adjacent charge states to be represented in the enumerated conformer set. Lenvatinib has neutral and anionic conformers but no cationic conformer corresponding to quinoline protonation, so the corresponding basic pK_a cannot be obtained from this set. Where multiple transitions are available, the transition closest to the sampled pH is reported; both crizotinib transitions are listed.

A pK_a obtained this way inherits the reference pH of the state penalties, because Equa-

tion S2 contains no pH term of its own. The Epik penalties used here were generated at pH 7.4, matching the pH of the calculations, so no offset is applied. The result is consequently a property of the protonation model that defines the solution reference, and not a result of the Poisson–Boltzmann or Monte Carlo steps. It is reported so that the reference state itself can be checked against experiment.

Trial-to-trial spread and the occupancy variance

Trial-to-trial variation is associated with the population balance at the sampled pH. For a binary state indicator X, with probability f of occupying one state,

$$\mathrm{Var}(X) = f(1-f), \tag{S6}$$

which is largest for an even population split and vanishes when either state saturates. Across the 38 ligand sites, $f(1-f)$, evaluated using the bound population, correlates with the trial-to-trial charge range (Spearman $\rho = 0.84$). Its mean is 0.015 for the 22 sites with identical bound charges across trials and 0.153 for the 16 sites that vary. Of the 3668 per-residue apo-to-holo comparisons, 3179 are identical across trials and 489 vary. Equality here refers to the precision of the saved outputs. These associations do not establish convergence or exclude sampling limitations. For sites with substantial variation, the per-trial range should be considered alongside the mean.

Ligand population changes on binding

We observe that kinase–inhibitor binding redistributes population among the low-energy states retained by the enumeration cutoff ($\Delta G < 6$ kcal/mol), with higher-energy states remaining minor contributors in the bound ensemble. Here the cutoff refers to the Epik state penalty relative to the lowest-energy solution state at pH 7.4; it does not refer to the relative energies of states in the bound ensemble. Among the retained states, those at least

2.8 kcal/mol above the lowest-energy solution state contribute at most 5.6% of the bound population at any site in any of the three trials. Conformer-resolved populations are given in Table S2.

Taken across the set, the bound ensemble charge is within 0.05 of its solution value at 17 of the 38 ligand sites and differs by more than 0.3 of a proton at 14 of them. Including seven additional sites with substantial tautomer redistribution, 21 of the 38 sites show appreciable changes in protonation or tautomer populations on binding, spanning 9 of the 18 inhibitors. Eleven sites change the dominant charge state outright: crizotinib from +2 to +1 in eight structures, gefitinib from neutral to +1 in 2ITO and 2ITZ, and cobimetinib from +1 to neutral in 4AN2. Cobimetinib in 4LMN, lapatinib in 1XKK and nilotinib in 3CS9 are instead driven to a near-even mixture. These counts use the trial-averaged charges and populations; the seven additional tautomer-change sites are bosutinib in 3UE4, imatinib in 2HYY and 3PYY, ponatinib at both sites in 3ZOS, and axitinib in 4AG8 and 4AGC. The conformer populations and per-trial charges are given in Tables S2 and S4, respectively.

Inhibitors

ABL (Tyrosine Protein Kinase)

(a) Bosutinib (DB8)

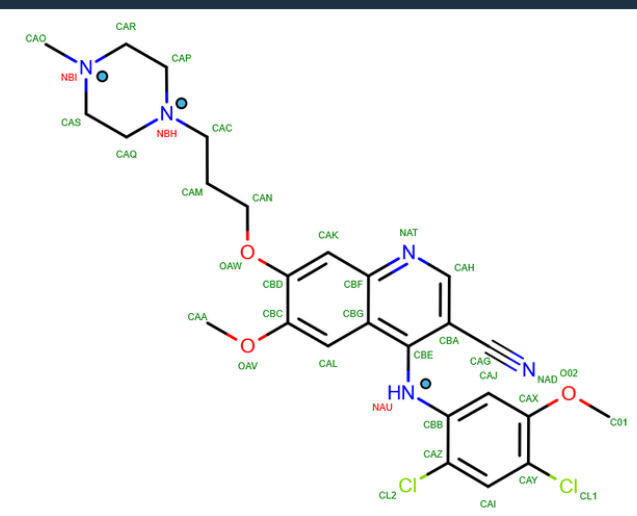

Conformer	Protonated Atoms	Charge	Epik Energy
DB8+1a	NAU, NBI	+1.0	0.071
DB8+1b	NAU, NBH	+1.0	1.695
DB8 0a	NAU	0.0	1.715
DB8 0b	NAU, NBI	0.0	5.065

Ensemble Charge: 0.944

ABL (Tyrosine Protein Kinase)

(b) Imatinib (STI)

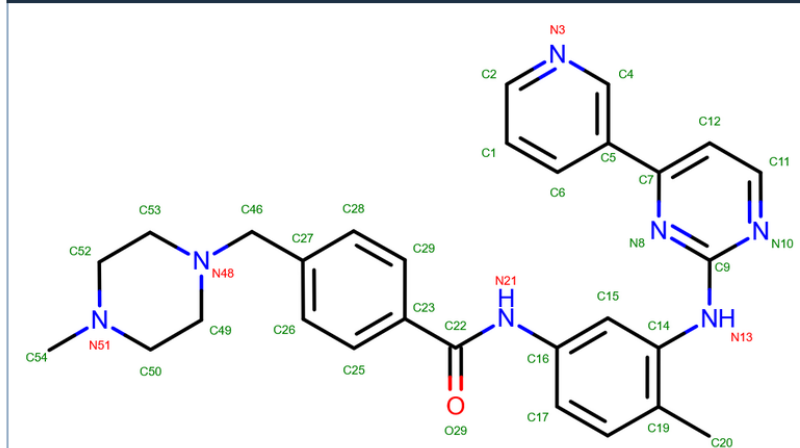

Conformer	Protonated Atoms	Charge	Epik Energy
STI+1a	N13, N21, N48	+1.0	0.316
STI+1b	N13, N21, N51	+1.0	0.573
STI 0	N13, N21	0.0	2.031
STI+2a	N13, N21, N48, N3	+2.0	5.430
STI+2b	N13, N21, N51, N3	+2.0	5.687

Ensemble Charge: 0.968

ABL (Tyrosine Protein Kinase)

(c) Nilotinib (NIL)

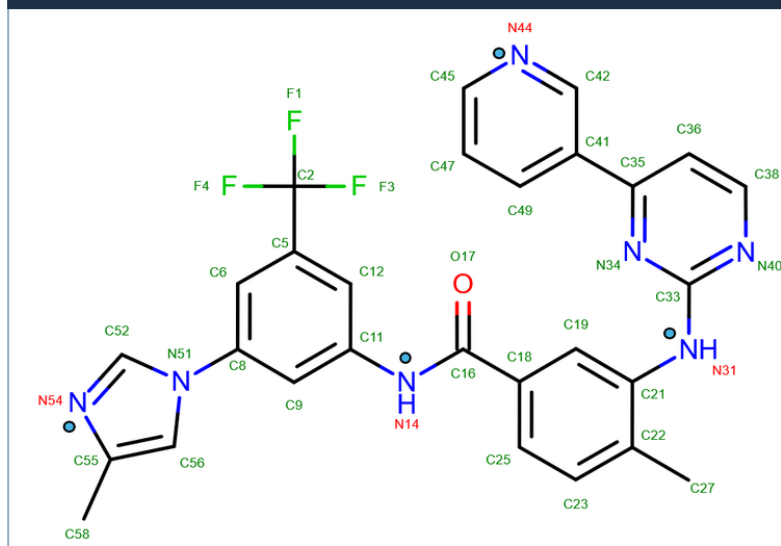

Conformer	Protonated Atoms	Charge	Epik Energy
NIL 0	N14, N31	0.0	0.058
NIL+1a	N14, N31, N54	+1.0	1.404
NIL+1b	N14, N31, N44	+1.0	5.173

Ensemble Charge: 0.094

ALK (Anaplastic Lymphoma Kinase)

(d) Alectinib (EMH)

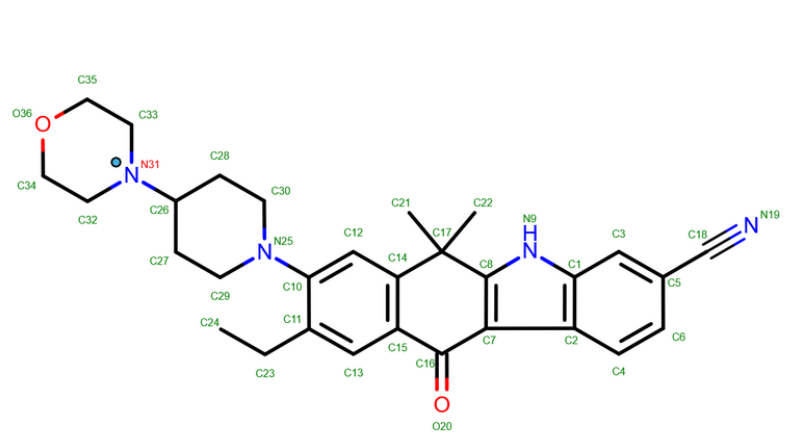

Conformer	Protonated Atoms	Charge	Epik Energy
EMH+1	N31, N9	+1.0	0.342
EMH 0	N9	0.0	0.488

Ensemble Charge: 0.561

ALK (Anaplastic Lymphoma Kinase)

(e) Ceritinib (4MK)

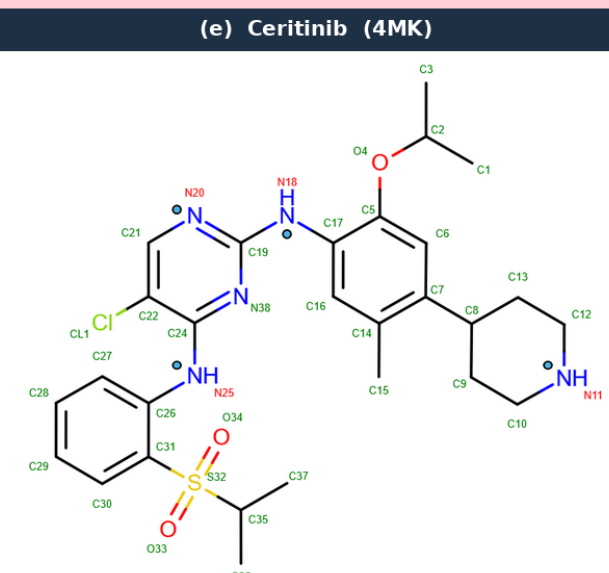

Conformer	Protonated Atoms	Charge	Epik Energy
4MK+1	N11, N18, N25	+1.0	0.000
4MK 0	N11, N18, N25	0.0	3.967
4MK+2	N11, N18, N20, N25	+2.0	5.439

Ensemble Charge: 0.999

ALK (Anaplastic Lymphoma Kinase)

(f) Crizotinib (VGH)

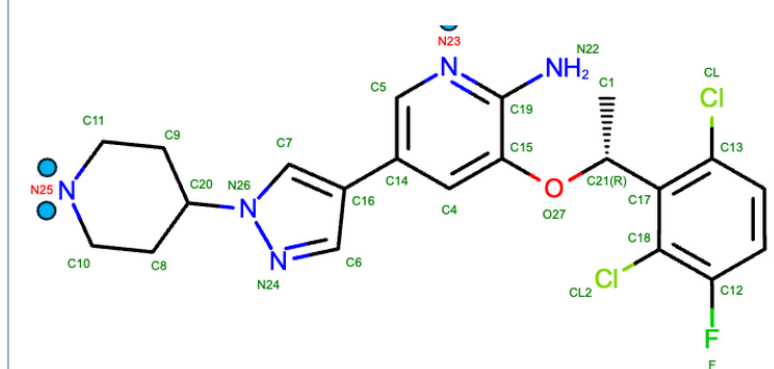

Conformer	Protonated Atoms	Charge	Epik Energy
VGH+1a	N22, N25	+1.0	0.585
VGH+1b	N22, N23, N25	+1.0	3.587
VGH+2	N22, N23, N25	+2.0	0.283
VGH 0	N22, N25	0.0	3.192

Ensemble Charge: 1.616

CDK6 (Cyclin-Dependent Kinase 6)

(g) Palbociclib (LQQ)

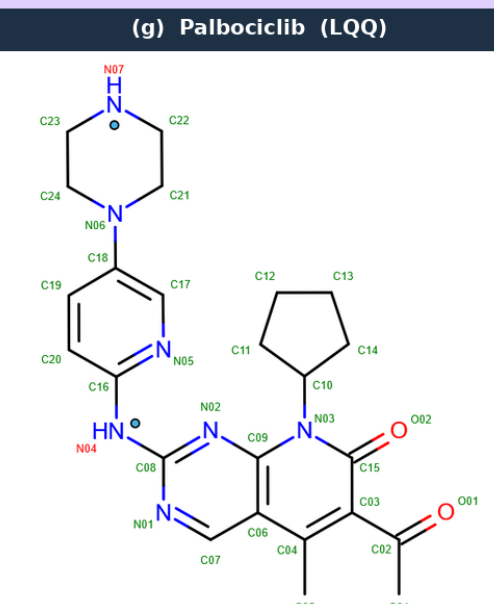

Conformer	Protonated Atoms	Charge	Epik Energy
LQQ+1	N04, N07	+1.0	0.256
LQQ 0	N04, N07	0.0	2.434

Ensemble Charge: 0.975

DDR1 (Discoidin Domain Receptor 1)

(h) Ponatinib (0LI)

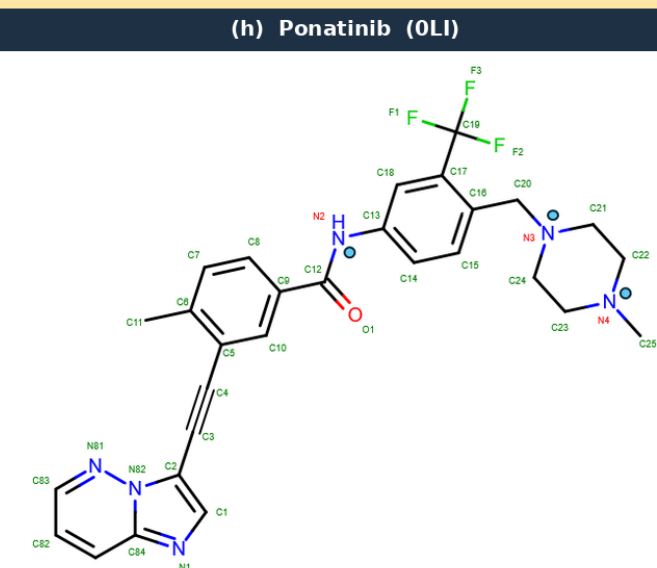

Conformer	Protonated Atoms	Charge	Epik Energy
0LI+1a	N2, N3	+1.0	0.316
0LI+1b	N2, N4	+1.0	0.573
0LI 0	N2	0.0	2.030

Ensemble Charge: 0.967

JAK3 (Janus Kinase 3)

(i) Tofacitinib (MI1)

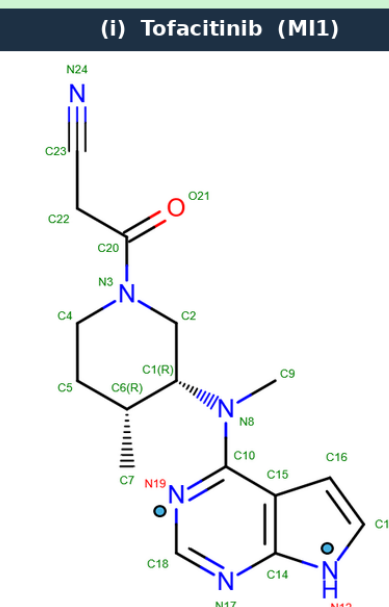

Conformer	Protonated Atoms	Charge	Epik Energy
MI1 0	N12	0.0	0.018
MI1+1	N12, N19	+1.0	2.096

Ensemble Charge: 0.029

Figure S1: Inhibitor conformer definitions, Epik energies, and ensemble charges. 2D chemical structures and protonation/tautomer states sampled for each kinase inhibitor in MCCE calculations, grouped by kinase target. Atom labels follow PDB naming conventions, and blue dots indicate sites at which protons may be added among the sampled conformers. Suffix notation (e.g., +1a, +1b) distinguishes alternative protonation/tautomer states of the same net charge. Epik energies are the total state penalties ΔG_{total} defined in the Methods, reported for each enumerated conformer, and ensemble charges are the statistical-mechanical averages obtained by Boltzmann-weighting them at pH 7.4. These values are identical in all three independent repeats of the pipeline, and per-trial values are listed in Table S4. Part 1: ABL inhibitors: (a) Bosutinib, (b) Imatinib, (c) Nilotinib; ALK inhibitors: (d) Alectinib, (e) Ceritinib, (f) Crizotinib; CDK6 inhibitor: (g) Palbociclib; DDR1 inhibitor: (h) Ponatinib; JAK3 inhibitor: (i) Tofacitinib

EGFR (Epidermal Growth Factor Receptor)

(j) Afatinib (0WN)

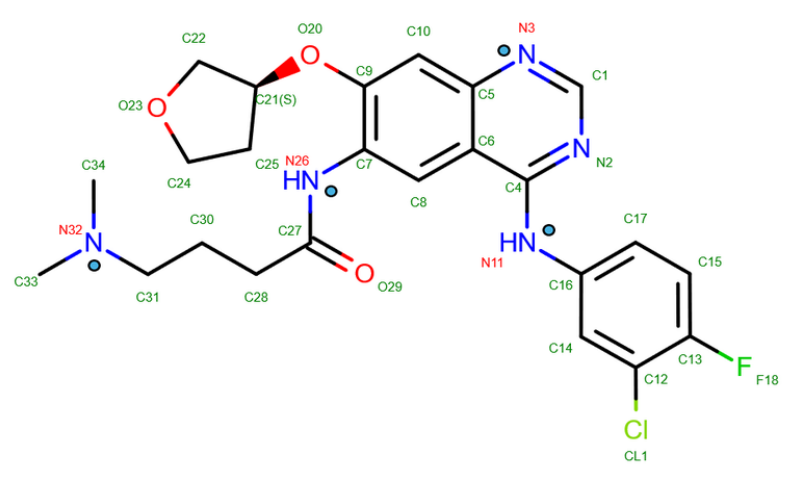

Conformer	Protonated Atoms	Charge	Epik Energy
0WN+1	N11, N26, N32	+1.0	0.005
0WN+2	N11, N26, N3, N32	+2.0	4.147
0WN 0	N11, N26	0.0	2.861

Ensemble Charge: 0.993

EGFR (Epidermal Growth Factor Receptor)

(k) Gefitinib (IRE)

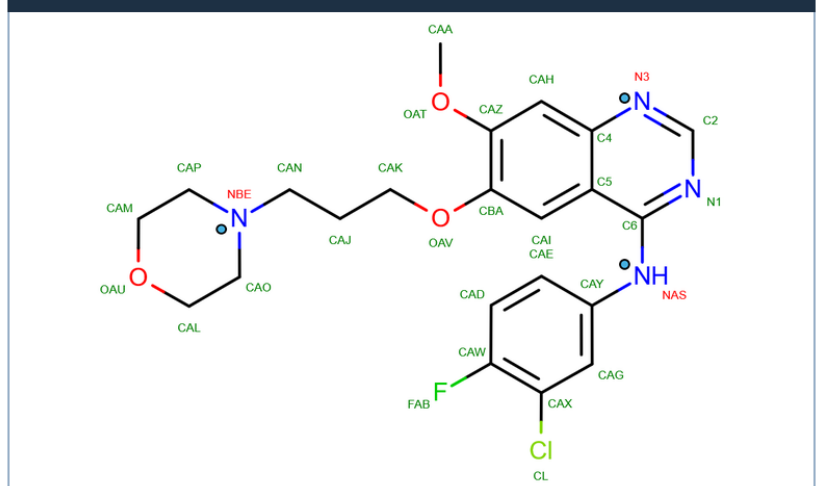

Conformer	Protonated Atoms	Charge	Epik Energy
IRE 0	NAS	0.0	0.212
IRE+1a	NAS, NBE	+1.0	0.717
IRE+1b	N3, NAS	+1.0	3.728
IRE+1c	N3, NAS	+1.0	4.395
IRE+2	N3, NAS, NBE	+2.0	4.412

Ensemble Charge: 0.302

EGFR (Epidermal Growth Factor Receptor)

(l) Lapatinib (FMM)

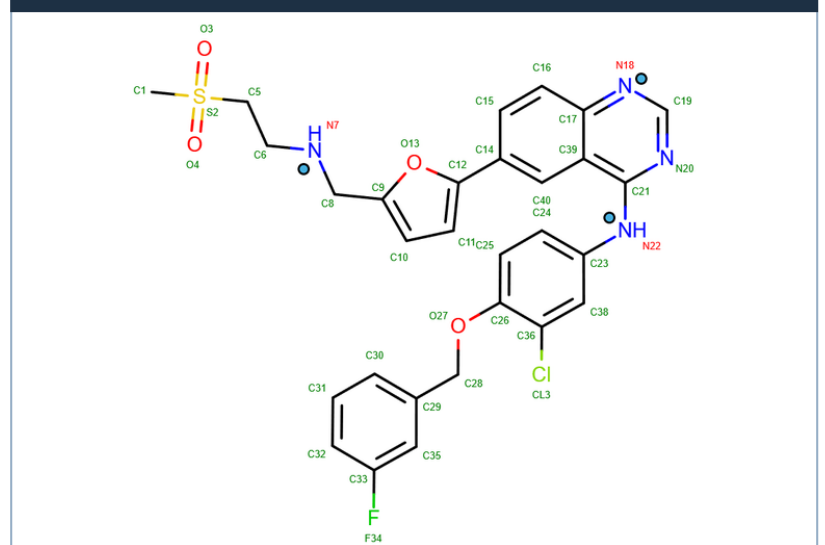

Conformer	Protonated Atoms	Charge	Epik Energy
FMM+1	N22, N7	+1.0	0.031
FMM 0	N22, N7	0.0	1.766
FMM+2	N18, N22, N7	+2.0	5.085

Ensemble Charge: 0.949

EGFR (Epidermal Growth Factor Receptor)

(m) Osimertinib (YY3)

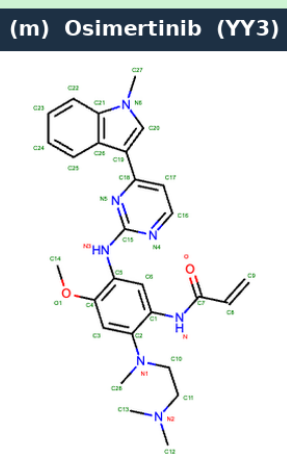

Conformer	Protonated Atoms	Charge	Epik Energy
YY3+1a	N, N2, N3	+1.0	0.016
YY3+1b	N, N1, N3	+1.0	3.310
YY3+1c	N2, N3, O	+1.0	3.893
YY3+1d	N1, N3, O	+1.0	4.431
YY3+2a	N, N2, N3, O	+2.0	4.972
YY3+2b	N, N1, N2, N3	+2.0	5.449
YY3 0	N, N3	0.0	2.299

Ensemble Charge: 0.980

MEK (Mitogen-Activated Protein Kinase Kinase)

(n) Cobimetinib (EUI)

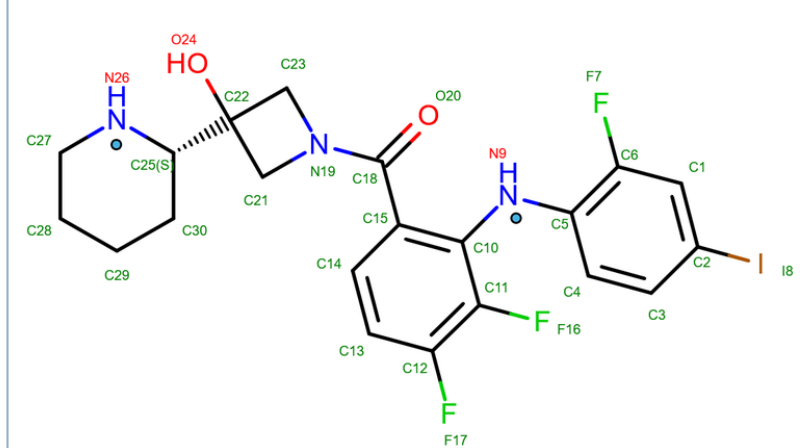

Conformer	Protonated Atoms	Charge	Epik Energy
EUI+1	N26, N9, O24	+1.0	0.008
EUI 0a	N26, N9, O24	0.0	2.536
EUI 0b	N26, N9, O24	0.0	5.637

Ensemble Charge: 0.986

VEGFR (Vascular Endothelial Growth Factor Receptor)

(o) Axitinib (AXI)

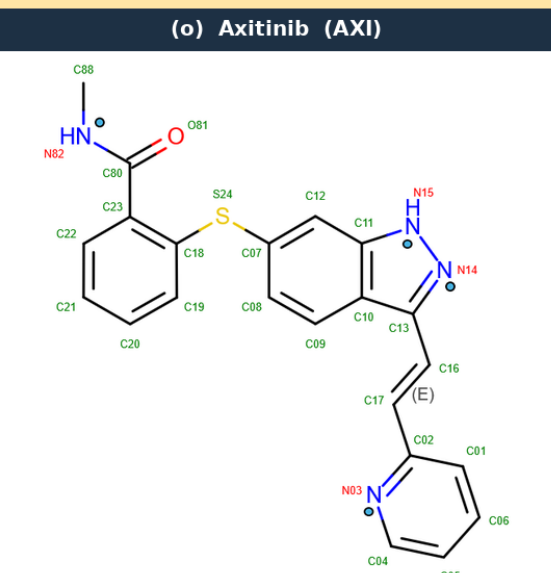

Conformer	Protonated Atoms	Charge	Epik Energy
AXI+1	N03, N15, N82	+1.0	3.790
AXI 0a	N15, N82	0.0	0.124
AXI 0b	N14, N82	0.0	0.993

Ensemble Charge: 0.002

VEGFR (Vascular Endothelial Growth Factor Receptor)

(p) Lenvatinib (LEV)

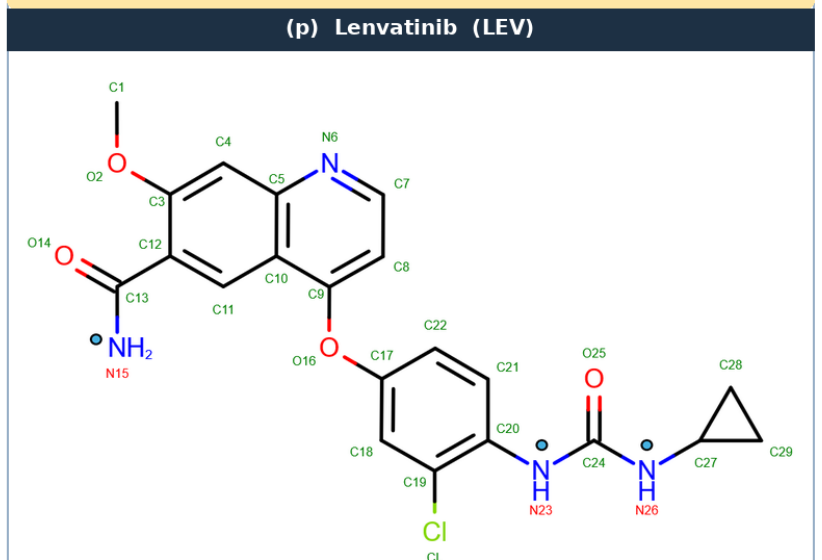

Conformer	Protonated Atoms	Charge	Epik Energy
LEV−1a	N15, N23, N26	−1.0	5.763
LEV−1b	N15, N23, N26	−1.0	5.763
LEV 0	N15, N23, N26	0.0	0.000

Ensemble Charge: 0.000

VEGFR (Vascular Endothelial Growth Factor Receptor)

(q) Sorafenib (BAX)

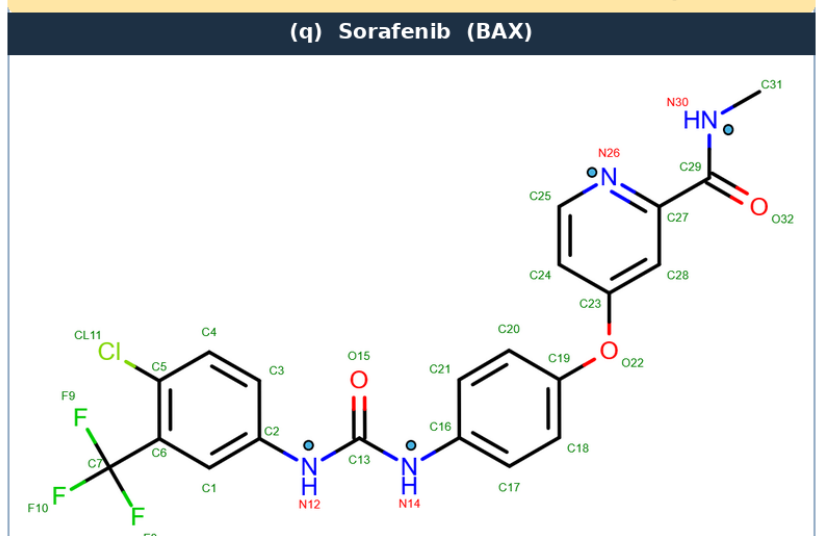

Conformer	Protonated Atoms	Charge	Epik Energy
BAX+1	N12, N14, N26, N30	+1.0	3.207
BAX−1	N12, N14	−1.0	5.840
BAX 0a	N12, N14, N30	0.0	0.004
BAX 0b	N12, N14, N26	0.0	3.620

Ensemble Charge: 0.004

VEGFR (Vascular Endothelial Growth Factor Receptor)

(r) Sunitinib (B49)

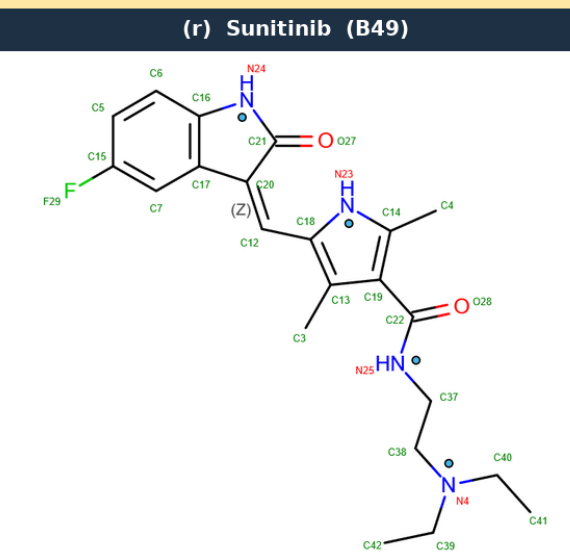

Conformer	Protonated Atoms	Charge	Epik Energy
B49+1	N23, N24, N25, N4	+1.0	0.008
B49 0a	N23, N24, N25	0.0	2.573
B49 0b	N23, N24, N4	0.0	5.676
B49 0c	N23, N25, N4	0.0	5.692

Ensemble Charge: 0.987

Figure S1 (continued): Inhibitor conformer definitions, Epik energies, and ensemble charges. Part 2: EGFR inhibitors: (j) Afatinib, (k) Gefitinib, (l) Lapatinib, (m) Osimertinib; MEK inhibitor: (n) Cobimetinib; VEGFR inhibitors: (o) Axitinib, (p) Lenvatinib, (q) Sorafenib, (r) Sunitinib.

Table S1: Kinase structures used in the MCCE calculations. Each calculation used the deposited coordinates as supplied, with no truncation, capping or loop repair; the residue ranges below are therefore those of the structure that was actually simulated. Chain: the chain retained from the deposited entry. Residues: the number of amino acids in the model. Ionizable: the number of Asp, Glu, Arg, Lys, His, Tyr and Cys residues, the set that can carry charge. Ponatinib occupies two sites in 3ZOS, which is a single structure and so appears once here.

Kinase	PDB ID	Chain	Residue range	Residues	Ionizable
ABL: ABL1 tyrosine protein kinase					
ABL	2HYY	A	235–498	263	91
ABL	3CS9	A	233–500	264	93
ABL	3PYY	A	251–519	265	94
ABL	3UE4	A	233–502	270	95
ABL	5MO4	A	83–531	429	146
ALK: Anaplastic lymphoma kinase					
ALK	2XP2	A	1093–1401	289	86
ALK	2YFX	A	1093–1401	293	87
ALK	3AOX	A	1086–1401	298	89
ALK	4ANQ	A	1093–1402	294	87
ALK	4ANS	A	1093–1402	294	87
ALK	4MKC	A	1093–1401	303	92
ALK	5AAA	A	1093–1401	295	88
ALK	5AAB	A	1093–1402	294	88
ALK	5AAC	A	1093–1402	293	88
CDK6: Cyclin-dependent kinase 6					
CDK6	2EUF	AB	8–305	526	160
CDK6	5L2I	A	10–301	260	85
DDR1: Discoidin domain receptor tyrosine kinase					
DDR1	3ZOS	A	602–913	301	92

Kinase	PDB ID	Chain	Residue range	Residues	Ionizable
EGFR: Epidermal growth factor receptor kinase					
EGFR	1XKK	A	702–1018	289	98
EGFR	2ITO	A	696–1019	303	104
EGFR	2ITY	A	697–1019	300	103
EGFR	2ITZ	A	697–1020	303	104
EGFR	3UG2	A	697–1018	299	106
EGFR	4G5J	A	696–1016	307	110
EGFR	4G5P	A	696–1016	299	104
EGFR	4I22	A	700–1014	297	103
EGFR	4WKQ	A	694–1020	296	100
EGFR	4ZAU	A	698–1017	283	98
JAK3: Janus kinase 3					
JAK3	3LXK	A	815–1098	279	93
MEK: Mitogen-activated protein kinase kinase					
MEK	4AN2	A	58–381	282	89
MEK	4LMN	A	61–382	292	90
MET: Tyrosine protein kinase Met					
MET	2WGJ	A	1052–1345	290	89
VEGFR: Vascular endothelial growth factor receptor kinase					
VEGFR	3WZD	A	820–1167	278	93
VEGFR	3WZE	A	815–1167	298	108
VEGFR	4AG8	A	816–1168	296	106
VEGFR	4AGC	A	801–1169	305	106
VEGFR	4AGD	A	802–1169	304	105
VEGFR	4ASD	A	807–1168	305	111

Table S2: Kinase inhibitor conformer charge states and MCCE calculated occupancies, averaged over three independent repeats. Results grouped by kinase type in bold. For each inhibitor (PDB structure) in italics, the conformer protonated atoms, Epik energy (the total state penalty ΔG_{total} defined in the Methods, kcal/mol) and net charge are listed, followed by $P(i)_{\mathrm{soln}}$, the MCCE calculated distribution in solution, $P(i)_{\mathrm{prot}}$, the distribution in the bound form, and the MCCE conformer name. Occupancies are means over all three trials with ±1 SEM. Uncertainties are rounded to three decimal places; a printed uncertainty of 0.000 does not necessarily imply identical trial values. Protonated atoms, Epik energy and net charge are properties of the ligand and do not vary between trials. The bold Ensemble row gives three ensemble charges, each the population-weighted mean $\sum_i q_i P(i)$ over the conformers of that inhibitor, evaluated under Charge with the Boltzmann-weighted Epik energies and under $P(i)_{\mathrm{soln}}$ and $P(i)_{\mathrm{prot}}$ with the MCCE sampled populations in solution and in the protein. The difference between the second and third is the shift produced by the binding site. Per-trial values for every structure are given in Table S4.

Conformer	Protonated atoms	Energy	Charge	$P(i)_{\mathrm{soln}}$	$P(i)_{\mathrm{prot}}$	MCCE
**ABL: ABL1 tyrosine protein kinase**						
Bosutinib (3UE4)						
DB8 0a	NAU	1.715	0.0	0.055 ± 0.000	0.046 ± 0.001	DB801
DB8 0b	NBI	5.065	0.0	0.000 ± 0.000	0.000 ± 0.000	DB802
DB8+1a	NAU, NBI	0.071	1.0	0.888 ± 0.000	0.566 ± 0.004	DB8+1
DB8+1b	NAU, NBH	1.695	1.0	0.057 ± 0.000	0.389 ± 0.003	DB8+2
**Ensemble**			**0.94**	**0.95**	**0.95**	
Imatinib (2HYY)						
STI 0	N13, N21	2.031	0.0	0.032 ± 0.000	0.000 ± 0.000	STI01
STI+1a	N13, N21, N48	0.316	1.0	0.588 ± 0.000	0.001 ± 0.001	STI+1
STI+1b	N13, N21, N51	0.573	1.0	0.380 ± 0.000	0.999 ± 0.001	STI+2
STI+2a	N13, N21, N49, N3	5.430	2.0	0.000 ± 0.000	0.000 ± 0.000	STI+a
STI+2b	N13, N21, N51, N3	5.687	2.0	0.000 ± 0.000	0.000 ± 0.000	STI+b
**Ensemble**			**0.97**	**0.97**	**1.00**	
Imatinib (3PYY)						
STI 0	N13, N21	2.031	0.0	0.032 ± 0.000	0.000 ± 0.000	STI01
STI+1a	N13, N21, N48	0.316	1.0	0.588 ± 0.000	0.004 ± 0.001	STI+1
STI+1b	N13, N21, N51	0.573	1.0	0.380 ± 0.000	0.996 ± 0.001	STI+2
STI+2a	N13, N21, N49, N3	5.430	2.0	0.000 ± 0.000	0.000 ± 0.000	STI+a

Continued on next page...

Continued from previous page

Conformer	Protonated atoms	Energy	Charge	$P(i)_{\text{soln}}$	$P(i)_{\text{prot}}$	MCCE
STI+2b	N13, N21, N51, N3	5.687	2.0	0.000 ± 0.000	0.000 ± 0.000	STI+b
**Ensemble**			**0.97**	**0.97**	**1.00**	
Nilotinib (3CS9)						
NIL 0	N14, N31	0.058	0.0	0.907 ± 0.000	0.587 ± 0.126	NIL01
NIL+1a	N14, N31, N54	1.404	1.0	0.093 ± 0.000	0.413 ± 0.126	NIL+1
NIL+1b	N14, N31, N44	5.173	1.0	0.000 ± 0.000	0.000 ± 0.000	NIL+2
**Ensemble**			**0.09**	**0.09**	**0.41**	
Nilotinib (5MO4)						
NIL 0	N14, N31	0.058	0.0	0.907 ± 0.000	0.766 ± 0.007	NIL01
NIL+1a	N14, N31, N54	1.404	1.0	0.093 ± 0.000	0.234 ± 0.007	NIL+1
NIL+1b	N14, N31, N44	5.173	1.0	0.000 ± 0.000	0.000 ± 0.000	NIL+2
**Ensemble**			**0.09**	**0.09**	**0.23**	
**ALK: Anaplastic lymphoma kinase**						
Alectinib (3AOX)						
EMH 0	—	0.488	0.0	0.439 ± 0.000	0.174 ± 0.037	EMH01
EMH+1	N31	0.342	1.0	0.561 ± 0.000	0.826 ± 0.037	EMH+1
**Ensemble**			**0.56**	**0.56**	**0.83**	
Ceritinib (4MKC)						
4MK 0	N11, N18, N25	3.967	0.0	0.001 ± 0.000	0.000 ± 0.000	4MK01
4MK+1	N11(2), N18, N25	0.000	1.0	0.999 ± 0.000	1.000 ± 0.000	4MK+1
4MK+2	N11(2), N18, N20, N25	5.439	2.0	0.000 ± 0.000	0.000 ± 0.000	4MK+a
**Ensemble**			**1.00**	**1.00**	**1.00**	
Crizotinib (2XP2)						
VGH 0	N22(2), N25	3.192	0.0	0.005 ± 0.000	0.000 ± 0.000	VGH01
VGH+1a	N22(2), N25(2)	0.585	1.0	0.378 ± 0.000	1.000 ± 0.000	VGH+1
VGH+1b	N22(2), N25, N23	3.587	1.0	0.002 ± 0.000	0.000 ± 0.000	VGH+2
VGH+2	N22(2), N25(2), N23	0.283	2.0	0.615 ± 0.000	0.000 ± 0.000	VGH+a
**Ensemble**			**1.62**	**1.61**	**1.00**	
Crizotinib (2YFX)						

Continued on next page...

Continued from previous page						
Conformer	Protonated atoms	Energy	Charge	$P(i)_{\text{soln}}$	$P(i)_{\text{prot}}$	MCCE
VGH 0	N22(2), N25	3.192	0.0	0.005 ± 0.000	0.000 ± 0.000	VGH01
VGH+1a	N22(2), N25(2)	0.585	1.0	0.378 ± 0.000	1.000 ± 0.000	VGH+1
VGH+1b	N22(2), N25, N23	3.587	1.0	0.002 ± 0.000	0.000 ± 0.000	VGH+2
VGH+2	N22(2), N25(2), N23	0.283	2.0	0.615 ± 0.000	0.000 ± 0.000	VGH+a
**Ensemble**			**1.62**	**1.61**	**1.00**	
Crizotinib (4ANQ)						
VGH 0	N22(2), N25	3.192	0.0	0.005 ± 0.000	0.000 ± 0.000	VGH01
VGH+1a	N22(2), N25(2)	0.585	1.0	0.378 ± 0.000	1.000 ± 0.000	VGH+1
VGH+1b	N22(2), N25, N23	3.587	1.0	0.002 ± 0.000	0.000 ± 0.000	VGH+2
VGH+2	N22(2), N25(2), N23	0.283	2.0	0.615 ± 0.000	0.000 ± 0.000	VGH+a
**Ensemble**			**1.62**	**1.61**	**1.00**	
Crizotinib (4ANS)						
VGH 0	N22(2), N25	3.192	0.0	0.005 ± 0.000	0.000 ± 0.000	VGH01
VGH+1a	N22(2), N25(2)	0.585	1.0	0.378 ± 0.000	1.000 ± 0.000	VGH+1
VGH+1b	N22(2), N25, N23	3.587	1.0	0.002 ± 0.000	0.000 ± 0.000	VGH+2
VGH+2	N22(2), N25(2), N23	0.283	2.0	0.615 ± 0.000	0.000 ± 0.000	VGH+a
**Ensemble**			**1.62**	**1.61**	**1.00**	
Crizotinib (5AAA)						
VGH 0	N22(2), N25	3.192	0.0	0.005 ± 0.000	0.000 ± 0.000	VGH01
VGH+1a	N22(2), N25(2)	0.585	1.0	0.378 ± 0.000	1.000 ± 0.000	VGH+1
VGH+1b	N22(2), N25, N23	3.587	1.0	0.002 ± 0.000	0.000 ± 0.000	VGH+2
VGH+2	N22(2), N25(2), N23	0.283	2.0	0.615 ± 0.000	0.000 ± 0.000	VGH+a
**Ensemble**			**1.62**	**1.61**	**1.00**	
Crizotinib (5AAB)						
VGH 0	N22(2), N25	3.192	0.0	0.005 ± 0.000	0.000 ± 0.000	VGH01
VGH+1a	N22(2), N25(2)	0.585	1.0	0.378 ± 0.000	1.000 ± 0.000	VGH+1
VGH+1b	N22(2), N25, N23	3.587	1.0	0.002 ± 0.000	0.000 ± 0.000	VGH+2
VGH+2	N22(2), N25(2), N23	0.283	2.0	0.615 ± 0.000	0.000 ± 0.000	VGH+a
**Ensemble**			**1.62**	**1.61**	**1.00**	

Continued on next page...

Continued from previous page						
Conformer	Protonated atoms	Energy	Charge	$P(i)_{\mathrm{soln}}$	$P(i)_{\mathrm{prot}}$	MCCE
**CDK6: Cyclin dependent kinase 6**						
Palbociclib (2EUF)						
LQQ 0	N4, N7(2)	2.434	0.0	0.025 ± 0.000	0.000 ± 0.000	LQQ01
LQQ+1	N4, N7	0.256	1.0	0.975 ± 0.000	1.000 ± 0.000	LQQ+1
**Ensemble**			**0.98**	**0.97**	**1.00**	
Palbociclib (5L2I)						
LQQ 0	N4, N7(2)	2.434	0.0	0.025 ± 0.000	0.006 ± 0.000	LQQ01
LQQ+1	N4, N7	0.256	1.0	0.975 ± 0.000	0.994 ± 0.000	LQQ+1
**Ensemble**			**0.98**	**0.97**	**0.99**	
**DDR1: Discoidin domain receptor tyrosine kinase**						
Ponatinib (3ZOS, buried†)						
0LI 0	N2	2.030	0.0	0.037 ± 0.000	0.000 ± 0.000	0LI01
0LI+1	N2, N3	0.316	1.0	0.598 ± 0.000	0.000 ± 0.000	0LI+1
0LI+2	N2, N4	0.573	1.0	0.365 ± 0.000	1.000 ± 0.000	0LI+2
**Ensemble**			**0.97**	**0.96**	**1.00**	
Ponatinib (3ZOS, surface†)						
0LI 0	N2	2.030	0.0	0.036 ± 0.000	0.104 ± 0.005	0LI01
0LI+1	N2, N3	0.316	1.0	0.589 ± 0.000	0.181 ± 0.011	0LI+1
0LI+2	N2, N4	0.573	1.0	0.375 ± 0.000	0.715 ± 0.013	0LI+2
**Ensemble**			**0.97**	**0.96**	**0.90**	
**EGFR: Epidermal growth factor receptor kinase**						
Afatinib (4G5J)						
0WN 0	N11, N26	2.861	0.0	0.008 ± 0.000	0.055 ± 0.001	0WN01
0WN+1	N11, N26, N32	0.005	1.0	0.992 ± 0.000	0.945 ± 0.001	0WN+1
0WN+2	N11, N26, N32, N3	4.147	2.0	0.000 ± 0.000	0.000 ± 0.000	0WN+a
**Ensemble**			**0.99**	**0.99**	**0.94**	
Afatinib (4G5P)						
0WN 0	N11, N26	2.861	0.0	0.008 ± 0.000	0.039 ± 0.000	0WN01
0WN+1	N11, N26, N32	0.005	1.0	0.992 ± 0.000	0.961 ± 0.000	0WN+1

Continued on next page...

Continued from previous page

Conformer	Protonated atoms	Energy	Charge	$P(i)_{\text{soln}}$	$P(i)_{\text{prot}}$	MCCE
0WN+2	N11, N26, N32, N3	4.147	2.0	0.000 ± 0.000	0.000 ± 0.000	0WN+a
**Ensemble**			**0.99**	**0.99**	**0.96**	
Gefitinib (2ITO)						
IRE 0	NAS	0.212	0.0	0.700 ± 0.000	0.217 ± 0.017	IRE01
IRE+1a	NAS, NBE	0.717	1.0	0.298 ± 0.000	0.783 ± 0.017	IRE+1
IRE+1b	NAS, N3	3.728	1.0	0.002 ± 0.000	0.000 ± 0.000	IRE+2
IRE+1c	NAS, N3	4.395	1.0	0.000 ± 0.000	0.000 ± 0.000	IRE+3
IRE+2	NAS, N3, NBE	4.412	2.0	0.000 ± 0.000	0.000 ± 0.000	IRE+a
**Ensemble**			**0.30**	**0.30**	**0.78**	
Gefitinib (2ITY)						
IRE 0	NAS	0.212	0.0	0.700 ± 0.000	0.650 ± 0.001	IRE01
IRE+1a	NAS, NBE	0.717	1.0	0.298 ± 0.000	0.350 ± 0.001	IRE+1
IRE+1b	NAS, N3	3.728	1.0	0.002 ± 0.000	0.000 ± 0.000	IRE+2
IRE+1c	NAS, N3	4.395	1.0	0.000 ± 0.000	0.000 ± 0.000	IRE+3
IRE+2	NAS, N3, NBE	4.412	2.0	0.000 ± 0.000	0.000 ± 0.000	IRE+a
**Ensemble**			**0.30**	**0.30**	**0.35**	
Gefitinib (2ITZ)						
IRE 0	NAS	0.212	0.0	0.700 ± 0.000	0.349 ± 0.007	IRE01
IRE+1a	NAS, NBE	0.717	1.0	0.298 ± 0.000	0.651 ± 0.007	IRE+1
IRE+1b	NAS, N3	3.728	1.0	0.002 ± 0.000	0.000 ± 0.000	IRE+2
IRE+1c	NAS, N3	4.395	1.0	0.000 ± 0.000	0.000 ± 0.000	IRE+3
IRE+2	NAS, N3, NBE	4.412	2.0	0.000 ± 0.000	0.000 ± 0.000	IRE+a
**Ensemble**			**0.30**	**0.30**	**0.65**	
Gefitinib (3UG2)						
IRE 0	NAS	0.212	0.0	0.700 ± 0.000	0.991 ± 0.005	IRE01
IRE+1a	NAS, NBE	0.717	1.0	0.298 ± 0.000	0.009 ± 0.005	IRE+1
IRE+1b	NAS, N3	3.728	1.0	0.002 ± 0.000	0.000 ± 0.000	IRE+2
IRE+1c	NAS, N3	4.395	1.0	0.000 ± 0.000	0.000 ± 0.000	IRE+3
IRE+2	NAS, N3, NBE	4.412	2.0	0.000 ± 0.000	0.000 ± 0.000	IRE+a

Continued on next page...

Continued from previous page						
Conformer	Protonated atoms	Energy	Charge	$P(i)_{\mathrm{soln}}$	$P(i)_{\mathrm{prot}}$	MCCE
**Ensemble**			**0.30**	**0.30**	**0.01**	
Gefitinib (4I22)						
IRE 0	NAS	0.212	0.0	0.700 ± 0.000	0.503 ± 0.013	IRE01
IRE+1a	NAS, NBE	0.717	1.0	0.298 ± 0.000	0.497 ± 0.013	IRE+1
IRE+1b	NAS, N3	3.728	1.0	0.002 ± 0.000	0.000 ± 0.000	IRE+2
IRE+1c	NAS, N3	4.395	1.0	0.000 ± 0.000	0.000 ± 0.000	IRE+3
IRE+2	NAS, N3, NBE	4.412	2.0	0.000 ± 0.000	0.000 ± 0.000	IRE+a
**Ensemble**			**0.30**	**0.30**	**0.50**	
Gefitinib (4WKQ)						
IRE 0	NAS	0.212	0.0	0.700 ± 0.000	0.538 ± 0.019	IRE01
IRE+1a	NAS, NBE	0.717	1.0	0.298 ± 0.000	0.462 ± 0.019	IRE+1
IRE+1b	NAS, N3	3.728	1.0	0.002 ± 0.000	0.000 ± 0.000	IRE+2
IRE+1c	NAS, N3	4.395	1.0	0.000 ± 0.000	0.000 ± 0.000	IRE+3
IRE+2	NAS, N3, NBE	4.412	2.0	0.000 ± 0.000	0.000 ± 0.000	IRE+a
**Ensemble**			**0.30**	**0.30**	**0.46**	
Lapatinib (1XKK)						
FMM 0	N7, N22	1.766	0.0	0.051 ± 0.000	0.424 ± 0.061	FMM01
FMM+1	N7(2), N22	0.031	1.0	0.949 ± 0.000	0.576 ± 0.061	FMM+1
FMM+2	N7(2), N22, N18	5.085	2.0	0.000 ± 0.000	0.000 ± 0.000	FMM+a
**Ensemble**			**0.95**	**0.95**	**0.58**	
Osimertinib (4ZAU)						
YY3 0	N, N3	2.299	0.0	0.021 ± 0.000	0.008 ± 0.001	YY301
YY3+1a	N, N2, N3	0.016	1.0	0.974 ± 0.000	0.985 ± 0.002	YY3+1
YY3+1b	N, N1, N3	3.310	1.0	0.004 ± 0.000	0.003 ± 0.001	YY3+2
YY3+1c	O, N2, N3	3.893	1.0	0.001 ± 0.000	0.004 ± 0.002	YY3+3
YY3+1d	O, N1, N3	4.431	1.0	0.000 ± 0.000	0.000 ± 0.000	YY3+4
YY3+2a	O, N, N2, N3	4.972	2.0	0.000 ± 0.000	0.000 ± 0.000	YY3+a
YY3+2b	N, N1, N2, N3	5.449	2.0	0.000 ± 0.000	0.000 ± 0.000	YY3+b
**Ensemble**			**0.98**	**0.98**	**0.99**	

Continued on next page...

Continued from previous page						
Conformer	Protonated atoms	Energy	Charge	$P(i)_{\mathrm{soln}}$	$P(i)_{\mathrm{prot}}$	MCCE
**JAK3: Janus kinase 3**						
Tofacitinib (3LXK)						
MI1 0	N12	0.018	0.0	0.971 ± 0.000	0.957 ± 0.003	MI101
MI1+1	N12, N19	2.096	1.0	0.029 ± 0.000	0.043 ± 0.003	MI1+1
**Ensemble**			**0.03**	**0.03**	**0.04**	
**MEK: Mitogen-activated protein kinase**						
Cobimetinib (4AN2)						
EUI 0a	O24, N9, N26	2.536	0.0	0.014 ± 0.000	0.777 ± 0.005	EUI01
EUI 0b	N9, N26(2)	5.637	0.0	0.000 ± 0.000	0.002 ± 0.000	EUI02
EUI+1	O24, N9, N26(2)	0.008	1.0	0.986 ± 0.000	0.222 ± 0.005	EUI+1
**Ensemble**			**0.99**	**0.99**	**0.22**	
Cobimetinib (4LMN)						
EUI 0a	O24, N9, N26	2.536	0.0	0.014 ± 0.000	0.478 ± 0.006	EUI01
EUI 0b	N9, N26(2)	5.637	0.0	0.000 ± 0.000	0.000 ± 0.000	EUI02
EUI+1	O24, N9, N26(2)	0.008	1.0	0.986 ± 0.000	0.522 ± 0.006	EUI+1
**Ensemble**			**0.99**	**0.99**	**0.52**	
**MET: Tyr protein kinase**						
Crizotinib (2WGJ)						
VGH 0	N22(2), N25	3.192	0.0	0.005 ± 0.000	0.039 ± 0.003	VGH01
VGH+1a	N22(2), N25(2)	0.585	1.0	0.378 ± 0.000	0.961 ± 0.003	VGH+1
VGH+1b	N22(2), N25, N23	3.587	1.0	0.002 ± 0.000	0.000 ± 0.000	VGH+2
VGH+2	N22(2), N25(2), N23	0.283	2.0	0.615 ± 0.000	0.000 ± 0.000	VGH+a
**Ensemble**			**1.62**	**1.61**	**0.96**	
**VEGFR: Vascular endothelial growth factor receptor kinase**						
Axitinib (4AG8)						
AXI 0a	N82, N15	0.124	0.0	0.810 ± 0.000	0.961 ± 0.000	AXI01
AXI 0b	N82, N14	0.993	0.0	0.187 ± 0.000	0.039 ± 0.000	AXI02
AXI+1	N3, N15, N82	3.790	1.0	0.003 ± 0.000	0.000 ± 0.000	AXI+1
**Ensemble**			**0.00**	**0.00**	**0.00**	

Continued on next page...

Continued from previous page						
Conformer	Protonated atoms	Energy	Charge	$P(i)_{\text{soln}}$	$P(i)_{\text{prot}}$	MCCE
Axitinib (4AGC)						
AXI 0a	N82, N15	0.124	0.0	0.810 ± 0.000	0.988 ± 0.001	AXI01
AXI 0b	N82, N14	0.993	0.0	0.187 ± 0.000	0.012 ± 0.001	AXI02
AXI+1	N3, N15, N82	3.790	1.0	0.003 ± 0.000	0.000 ± 0.000	AXI+1
**Ensemble**			**0.00**	**0.00**	**0.00**	
Lenvatinib (3WZD)						
LEV 0	N15(2), N23, N26	0.000	0.0	1.000 ± 0.000	1.000 ± 0.000	LEV01
LEV-1a	N15, N23, N26	5.763	-1.0	0.000 ± 0.000	0.000 ± 0.000	LEV-1
LEV-1b	N15, N23, N26	5.763	-1.0	0.000 ± 0.000	0.000 ± 0.000	LEV-2
**Ensemble**			**-0.00**	**0.00**	**0.00**	
Sorafenib (3WZE)						
BAX 0a	N12, N14, N30	0.004	0.0	0.994 ± 0.000	1.000 ± 0.000	BAX01
BAX+1	N12, N14, N26, N30	3.207	1.0	0.004 ± 0.000	0.000 ± 0.000	BAX+1
BAX 0b	N12, N14, N26	3.620	0.0	0.002 ± 0.000	0.000 ± 0.000	BAX02
BAX-1	N12, N14	5.840	-1.0	0.000 ± 0.000	0.000 ± 0.000	BAX-1
**Ensemble**			**0.00**	**0.00**	**0.00**	
Sorafenib (4ASD)						
BAX 0a	N12, N14, N30	0.004	0.0	0.994 ± 0.000	1.000 ± 0.000	BAX01
BAX+1	N12, N14, N26, N30	3.207	1.0	0.004 ± 0.000	0.000 ± 0.000	BAX+1
BAX 0b	N12, N14, N26	3.620	0.0	0.002 ± 0.000	0.000 ± 0.000	BAX02
BAX-1	N12, N14	5.840	-1.0	0.000 ± 0.000	0.000 ± 0.000	BAX-1
**Ensemble**			**0.00**	**0.00**	**0.00**	
Sunitinib (4AGD)						
B49 0a	N23, N24, N25	2.573	0.0	0.013 ± 0.000	0.101 ± 0.011	B4901
B49 0b	N4, N23, N24	5.676	0.0	0.000 ± 0.000	0.000 ± 0.000	B4902
B49 0c	N4, N23, N25	5.692	0.0	0.000 ± 0.000	0.000 ± 0.000	B4903
B49+1	N4, N23, N24, N25	0.008	1.0	0.987 ± 0.000	0.899 ± 0.011	B49+1
**Ensemble**			**0.99**	**0.99**	**0.90**	

†Ponatinib binds at two distinct sites in the 3ZOS crystal structure: a buried active site and a solvent-

exposed surface site, each with independent MCCE sampling.

Table S3: Residues whose protonation changes on inhibitor binding, averaged over three independent repeats. Tier is assigned on $|\Delta q|$: outlier ≥ 0.5, affected $0.2 \leq |\Delta q| < 0.5$. ΔCharge is the holo$-$apo difference computed within each trial and then averaged, with ± 1 SEM.

Tier	PDB	Inhibitor	ResName	Chain	ResNum	ΔCharge	±SEM
outlier	4MKC	Ceritinib	LYS	A	1205	−0.510	0.000
affected	1XKK	Lapatinib	LYS	A	745	−0.397	0.022
affected	1XKK	Lapatinib	CYS	A	775	+0.263	0.037
affected	3UE4	Bosutinib	GLU	A	286	−0.250	0.012
affected	4AN2	Cobimetinib	CYS	A	207	−0.230	0.006

Of the five residues in Table S3, four reproduce their tier assignment in all three trials. The exception is CYS^{-}A0775 in 1XKK, which gives +0.30, +0.19 and +0.30 across Trial01–03 and should therefore be read as a borderline case between the unaffected and affected categories.

Table S4: Per-trial values for charges of isolated ligands, apo-kinase and changes on binding, over three independent repeats of the full MCCE pipeline. Each trial is a complete, independent rerun of MCCE steps 1–4 from the same starting structure with its own Monte Carlo seed (Trial01–03 use `MONTE_SEED` 1001, 1002 and 1003). SEM is the standard error of the mean over the three trials, rounded to three decimal places. The bound ligand charge is classified as *identical* when all three trials return the same value at the saved output precision, and *variable* otherwise. These labels describe trial-to-trial variation and do not establish the number of underlying population modes. $\Delta crg_{Holo\text{-}Apo}$ is formed within each trial before averaging. Ponatinib occupies two sites in 3ZOS; the protein quantities are reported once, on the buried site.

PDBID	Ligand	Quantity	Trial01	Trial02	Trial03	Mean	SEM	Class
**ABL: ABL1 tyrosine protein kinase**								
3UE4	Bosutinib	crg_{soln}	0.940	0.940	0.940	0.940	0.000	
		crg_{bound}	0.950	0.950	0.950	0.950	0.000	identical
		crg_{Apo}	-4.570	-4.650	-4.520	-4.580	0.038	
		crg_{Holo}	-4.890	-4.960	-4.830	-4.893	0.038	
		$\Delta crg_{Holo\text{-}Apo}$	-0.320	-0.310	-0.310	-0.313	0.003	
2HYY	Imatinib	crg_{soln}	0.970	0.970	0.970	0.970	0.000	
		crg_{bound}	1.000	1.000	1.000	1.000	0.000	identical
		crg_{Apo}	-6.200	-6.180	-6.190	-6.190	0.006	
		crg_{Holo}	-6.400	-6.340	-6.380	-6.373	0.018	
		$\Delta crg_{Holo\text{-}Apo}$	-0.200	-0.160	-0.190	-0.183	0.012	
3PYY	Imatinib	crg_{soln}	0.970	0.970	0.970	0.970	0.000	
		crg_{bound}	1.000	1.000	1.000	1.000	0.000	identical
		crg_{Apo}	-4.800	-4.810	-4.810	-4.807	0.003	
		crg_{Holo}	-4.840	-4.850	-4.850	-4.847	0.003	
		$\Delta crg_{Holo\text{-}Apo}$	-0.040	-0.040	-0.040	-0.040	0.000	
3CS9	Nilotinib	crg_{soln}	0.090	0.090	0.090	0.090	0.000	
		crg_{bound}	0.420	0.630	0.190	0.413	0.127	variable
		crg_{Apo}	-4.620	-4.580	-4.610	-4.603	0.012	
		crg_{Holo}	-4.660	-4.610	-4.610	-4.627	0.017	
		$\Delta crg_{Holo\text{-}Apo}$	-0.040	-0.030	0.000	-0.023	0.012	
5MO4	Nilotinib	crg_{soln}	0.090	0.090	0.090	0.090	0.000	

Continued on next page...

Continued from previous page

PDBID	Ligand	Quantity	Trial01	Trial02	Trial03	Mean	SEM	Class
		crg_{bound}	0.230	0.230	0.250	0.237	0.007	variable
		crg_{Apo}	-7.970	-7.890	-7.950	-7.937	0.024	
		crg_{Holo}	-8.000	-7.890	-7.950	-7.947	0.032	
		$\Delta crg_{Holo\text{-}Apo}$	-0.030	0.000	0.000	-0.010	0.010	
**ALK: Anaplastic lymphoma kinase**								
3AOX	Alectinib	crg_{soln}	0.560	0.560	0.560	0.560	0.000	
		crg_{bound}	0.790	0.900	0.790	0.827	0.037	variable
		crg_{Apo}	-8.400	-8.370	-8.360	-8.377	0.012	
		crg_{Holo}	-8.440	-8.400	-8.420	-8.420	0.012	
		$\Delta crg_{Holo\text{-}Apo}$	-0.040	-0.030	-0.060	-0.043	0.009	
4MKC	Ceritinib	crg_{soln}	1.000	1.000	1.000	1.000	0.000	
		crg_{bound}	1.000	1.000	1.000	1.000	0.000	identical
		crg_{Apo}	-4.170	-4.180	-3.820	-4.057	0.118	
		crg_{Holo}	-4.740	-4.780	-4.430	-4.650	0.111	
		$\Delta crg_{Holo\text{-}Apo}$	-0.570	-0.600	-0.610	-0.593	0.012	
2XP2	Crizotinib	crg_{soln}	1.610	1.610	1.610	1.610	0.000	
		crg_{bound}	1.000	1.000	1.000	1.000	0.000	identical
		crg_{Apo}	-5.510	-5.960	-5.920	-5.797	0.144	
		crg_{Holo}	-5.580	-6.010	-5.990	-5.860	0.140	
		$\Delta crg_{Holo\text{-}Apo}$	-0.070	-0.050	-0.070	-0.063	0.007	
2YFX	Crizotinib	crg_{soln}	1.610	1.610	1.610	1.610	0.000	
		crg_{bound}	1.000	1.000	1.000	1.000	0.000	identical
		crg_{Apo}	-4.590	-4.590	-4.590	-4.590	0.000	
		crg_{Holo}	-4.590	-4.620	-4.610	-4.607	0.009	
		$\Delta crg_{Holo\text{-}Apo}$	0.000	-0.030	-0.020	-0.017	0.009	
4ANQ	Crizotinib	crg_{soln}	1.610	1.610	1.610	1.610	0.000	
		crg_{bound}	1.000	1.000	1.000	1.000	0.000	identical
		crg_{Apo}	-5.990	-5.410	-5.430	-5.610	0.190	
		crg_{Holo}	-6.030	-5.460	-5.490	-5.660	0.185	

Continued on next page...

PDBID	Ligand	Quantity	Trial01	Trial02	Trial03	Mean	SEM	Class
Continued from previous page								
		$\Delta crg_{Holo\text{-}Apo}$	-0.040	-0.050	-0.060	-0.050	0.006	
4ANS	Crizotinib	crg_{soln}	1.610	1.610	1.610	1.610	0.000	
		crg_{bound}	1.000	1.000	1.000	1.000	0.000	identical
		crg_{Apo}	-5.910	-5.360	-5.930	-5.733	0.187	
		crg_{Holo}	-5.950	-5.410	-5.990	-5.783	0.187	
		$\Delta crg_{Holo\text{-}Apo}$	-0.040	-0.050	-0.060	-0.050	0.006	
5AAA	Crizotinib	crg_{soln}	1.610	1.610	1.610	1.610	0.000	
		crg_{bound}	1.000	1.000	1.000	1.000	0.000	identical
		crg_{Apo}	-5.450	-5.460	-5.380	-5.430	0.025	
		crg_{Holo}	-5.520	-5.490	-5.440	-5.483	0.023	
		$\Delta crg_{Holo\text{-}Apo}$	-0.070	-0.030	-0.060	-0.053	0.012	
5AAB	Crizotinib	crg_{soln}	1.610	1.610	1.610	1.610	0.000	
		crg_{bound}	1.000	1.000	1.000	1.000	0.000	identical
		crg_{Apo}	-5.950	-6.350	-6.360	-6.220	0.135	
		crg_{Holo}	-6.020	-6.410	-6.420	-6.283	0.132	
		$\Delta crg_{Holo\text{-}Apo}$	-0.070	-0.060	-0.060	-0.063	0.003	
5AAC	Crizotinib	crg_{soln}	1.610	1.610	1.610	1.610	0.000	
		crg_{bound}	1.000	1.000	1.000	1.000	0.000	identical
		crg_{Apo}	-5.320	-5.790	-5.770	-5.627	0.153	
		crg_{Holo}	-5.400	-5.870	-5.860	-5.710	0.155	
		$\Delta crg_{Holo\text{-}Apo}$	-0.080	-0.080	-0.090	-0.083	0.003	
**CDK6: Cyclin-dependent kinase 6**								
2EUF	Palbociclib	crg_{soln}	0.980	0.980	0.980	0.980	0.000	
		crg_{bound}	1.000	1.000	1.000	1.000	0.000	identical
		crg_{Apo}	-9.330	-9.680	-9.750	-9.587	0.130	
		crg_{Holo}	-9.500	-9.860	-9.920	-9.760	0.131	
		$\Delta crg_{Holo\text{-}Apo}$	-0.170	-0.180	-0.170	-0.173	0.003	
5L2I	Palbociclib	crg_{soln}	0.980	0.980	0.980	0.980	0.000	
		crg_{bound}	0.990	0.990	0.990	0.990	0.000	identical

Continued on next page...

Continued from previous page								
PDBID	Ligand	Quantity	Trial01	Trial02	Trial03	Mean	SEM	Class
		crg_{Apo}	-0.190	-0.210	-0.300	-0.233	0.034	
		crg_{Holo}	-0.120	-0.230	-0.280	-0.210	0.047	
		$\Delta crg_{Holo\text{-}Apo}$	0.070	-0.020	0.020	0.023	0.026	
**DDR1: Discoidin domain receptor tyrosine kinase**								
3ZOS(b)	Ponatinib	crg_{soln}	0.960	0.960	0.960	0.960	0.000	
		crg_{bound}	1.000	1.000	1.000	1.000	0.000	identical
		crg_{Apo}	-1.640	-1.640	-1.660	-1.647	0.007	
		crg_{Holo}	-1.680	-1.690	-1.690	-1.687	0.003	
		$\Delta crg_{Holo\text{-}Apo}$	-0.040	-0.050	-0.030	-0.040	0.006	
3ZOS(s)	Ponatinib	crg_{soln}	0.960	0.960	0.960	0.960	0.000	
		crg_{bound}	0.900	0.900	0.890	0.897	0.003	variable
**EGFR: Epidermal growth factor receptor kinase**								
4G5J	Afatinib	crg_{soln}	0.990	0.990	0.990	0.990	0.000	
		crg_{bound}	0.950	0.940	0.950	0.947	0.003	variable
		crg_{Apo}	-6.450	-6.540	-6.420	-6.470	0.036	
		crg_{Holo}	-6.580	-6.630	-6.580	-6.597	0.017	
		$\Delta crg_{Holo\text{-}Apo}$	-0.130	-0.090	-0.160	-0.127	0.020	
4G5P	Afatinib	crg_{soln}	0.990	0.990	0.990	0.990	0.000	
		crg_{bound}	0.960	0.960	0.960	0.960	0.000	identical
		crg_{Apo}	-0.650	-0.570	-0.620	-0.613	0.023	
		crg_{Holo}	-0.950	-0.910	-0.930	-0.930	0.012	
		$\Delta crg_{Holo\text{-}Apo}$	-0.300	-0.340	-0.310	-0.317	0.012	
2ITO	Gefitinib	crg_{soln}	0.300	0.300	0.300	0.300	0.000	
		crg_{bound}	0.750	0.800	0.800	0.783	0.017	variable
		crg_{Apo}	-7.000	-7.130	-7.100	-7.077	0.039	
		crg_{Holo}	-7.150	-7.310	-7.320	-7.260	0.055	
		$\Delta crg_{Holo\text{-}Apo}$	-0.150	-0.180	-0.220	-0.183	0.020	
2ITY	Gefitinib	crg_{soln}	0.300	0.300	0.300	0.300	0.000	
		crg_{bound}	0.350	0.350	0.350	0.350	0.000	identical
								Continued on next page...

Continued from previous page								
PDBID	Ligand	Quantity	Trial01	Trial02	Trial03	Mean	SEM	Class
		crg_{Apo}	-6.050	-6.010	-6.030	-6.030	0.012	
		crg_{Holo}	-6.120	-6.090	-6.120	-6.110	0.010	
		$\Delta crg_{Holo\text{-}Apo}$	-0.070	-0.080	-0.090	-0.080	0.006	
2ITZ	Gefitinib	crg_{soln}	0.300	0.300	0.300	0.300	0.000	
		crg_{bound}	0.640	0.650	0.660	0.650	0.006	variable
		crg_{Apo}	-4.690	-4.980	-4.950	-4.873	0.092	
		crg_{Holo}	-4.790	-5.100	-5.040	-4.977	0.095	
		$\Delta crg_{Holo\text{-}Apo}$	-0.100	-0.120	-0.090	-0.103	0.009	
3UG2	Gefitinib	crg_{soln}	0.300	0.300	0.300	0.300	0.000	
		crg_{bound}	0.000	0.010	0.010	0.007	0.003	variable
		crg_{Apo}	-2.990	-3.030	-3.010	-3.010	0.012	
		crg_{Holo}	-2.950	-2.980	-2.930	-2.953	0.015	
		$\Delta crg_{Holo\text{-}Apo}$	0.040	0.050	0.080	0.057	0.012	
4I22	Gefitinib	crg_{soln}	0.300	0.300	0.300	0.300	0.000	
		crg_{bound}	0.480	0.490	0.520	0.497	0.012	variable
		crg_{Apo}	1.950	1.910	1.910	1.923	0.013	
		crg_{Holo}	1.930	1.810	1.810	1.850	0.040	
		$\Delta crg_{Holo\text{-}Apo}$	-0.020	-0.100	-0.100	-0.073	0.027	
4WKQ	Gefitinib	crg_{soln}	0.300	0.300	0.300	0.300	0.000	
		crg_{bound}	0.430	0.490	0.470	0.463	0.018	variable
		crg_{Apo}	-1.350	-1.340	-1.370	-1.353	0.009	
		crg_{Holo}	-1.380	-1.320	-1.380	-1.360	0.020	
		$\Delta crg_{Holo\text{-}Apo}$	-0.030	0.020	-0.010	-0.007	0.015	
1XKK	Lapatinib	crg_{soln}	0.950	0.950	0.950	0.950	0.000	
		crg_{bound}	0.700	0.500	0.530	0.577	0.062	variable
		crg_{Apo}	0.470	0.450	0.510	0.477	0.018	
		crg_{Holo}	0.020	0.110	0.130	0.087	0.034	
		$\Delta crg_{Holo\text{-}Apo}$	-0.450	-0.340	-0.380	-0.390	0.032	
4ZAU	Osimertinib	crg_{soln}	0.980	0.980	0.980	0.980	0.000	

Continued on next page...

Continued from previous page								
PDBID	Ligand	Quantity	Trial01	Trial02	Trial03	Mean	SEM	Class
		crg_{bound}	0.990	0.990	0.990	0.990	0.000	identical
		crg_{Apo}	-2.110	-2.140	-2.140	-2.130	0.010	
		crg_{Holo}	-2.420	-2.450	-2.460	-2.443	0.012	
		$\Delta crg_{Holo\text{-}Apo}$	-0.310	-0.310	-0.320	-0.313	0.003	
**JAK3: Janus kinase 3**								
3LXK	Tofacitinib	crg_{soln}	0.030	0.030	0.030	0.030	0.000	
		crg_{bound}	0.050	0.040	0.050	0.047	0.003	variable
		crg_{Apo}	1.140	1.290	1.100	1.177	0.058	
		crg_{Holo}	1.240	1.360	1.170	1.257	0.055	
		$\Delta crg_{Holo\text{-}Apo}$	0.100	0.070	0.070	0.080	0.010	
**MEK: Mitogen-activated protein kinase**								
4AN2	Cobimetinib	crg_{soln}	0.990	0.990	0.990	0.990	0.000	
		crg_{bound}	0.230	0.220	0.220	0.223	0.003	variable
		crg_{Apo}	-3.470	-3.530	-3.550	-3.517	0.024	
		crg_{Holo}	-3.640	-3.680	-3.670	-3.663	0.012	
		$\Delta crg_{Holo\text{-}Apo}$	-0.170	-0.150	-0.120	-0.147	0.015	
4LMN	Cobimetinib	crg_{soln}	0.990	0.990	0.990	0.990	0.000	
		crg_{bound}	0.510	0.530	0.520	0.520	0.006	variable
		crg_{Apo}	-3.600	-3.600	-3.500	-3.567	0.033	
		crg_{Holo}	-3.640	-3.600	-3.500	-3.580	0.042	
		$\Delta crg_{Holo\text{-}Apo}$	-0.040	0.000	0.000	-0.013	0.013	
**MET: Tyrosine protein kinase Met**								
2WGJ	Crizotinib	crg_{soln}	1.610	1.610	1.610	1.610	0.000	
		crg_{bound}	0.960	0.960	0.970	0.963	0.003	variable
		crg_{Apo}	7.000	7.020	7.050	7.023	0.015	
		crg_{Holo}	6.750	6.780	6.810	6.780	0.017	
		$\Delta crg_{Holo\text{-}Apo}$	-0.250	-0.240	-0.240	-0.243	0.003	
**VEGFR: Vascular endothelial growth factor receptor kinase**								
4AG8	Axitinib	crg_{soln}	0.000	0.000	0.000	0.000	0.000	

Continued on next page...

Continued from previous page								
PDBID	Ligand	Quantity	Trial01	Trial02	Trial03	Mean	SEM	Class
		crg_{bound}	0.000	0.000	0.000	0.000	0.000	identical
		crg_{Apo}	4.590	4.570	4.580	4.580	0.006	
		crg_{Holo}	4.620	4.600	4.620	4.613	0.007	
		$\Delta crg_{Holo\text{-}Apo}$	0.030	0.030	0.040	0.033	0.003	
4AGC	Axitinib	crg_{soln}	0.000	0.000	0.000	0.000	0.000	
		crg_{bound}	0.000	0.000	0.000	0.000	0.000	identical
		crg_{Apo}	3.430	3.550	3.500	3.493	0.035	
		crg_{Holo}	3.450	3.550	3.500	3.500	0.029	
		$\Delta crg_{Holo\text{-}Apo}$	0.020	0.000	0.000	0.007	0.007	
3WZD	Lenvatinib	crg_{soln}	-0.000	-0.000	-0.000	0.000	0.000	
		crg_{bound}	-0.000	-0.000	-0.000	0.000	0.000	identical
		crg_{Apo}	1.610	1.540	1.530	1.560	0.025	
		crg_{Holo}	1.630	1.570	1.540	1.580	0.026	
		$\Delta crg_{Holo\text{-}Apo}$	0.020	0.030	0.010	0.020	0.006	
3WZE	Sorafenib	crg_{soln}	0.000	0.000	0.000	0.000	0.000	
		crg_{bound}	0.000	0.000	0.000	0.000	0.000	identical
		crg_{Apo}	5.580	5.440	5.510	5.510	0.040	
		crg_{Holo}	5.590	5.420	5.500	5.503	0.049	
		$\Delta crg_{Holo\text{-}Apo}$	0.010	-0.020	-0.010	-0.007	0.009	
4ASD	Sorafenib	crg_{soln}	0.000	0.000	0.000	0.000	0.000	
		crg_{bound}	0.000	0.000	0.000	0.000	0.000	identical
		crg_{Apo}	0.260	0.120	0.290	0.223	0.052	
		crg_{Holo}	0.280	0.120	0.270	0.223	0.052	
		$\Delta crg_{Holo\text{-}Apo}$	0.020	0.000	-0.020	0.000	0.012	
4AGD	Sunitinib	crg_{soln}	0.990	0.990	0.990	0.990	0.000	
		crg_{bound}	0.890	0.920	0.890	0.900	0.010	variable
		crg_{Apo}	2.530	2.540	2.530	2.533	0.003	
		crg_{Holo}	2.440	2.460	2.420	2.440	0.012	
		$\Delta crg_{Holo\text{-}Apo}$	-0.090	-0.080	-0.110	-0.093	0.009	

Table S5: Differential binding free energies between tautomers of the same inhibitor. For two states i and j of the same ligand, $\Delta\Delta G$ is computed as defined in the Methods of the main text, with each row comparing two tautomers of the same net charge. $\Delta\Delta G$ is evaluated within each of the three trials and then averaged. The uncertainty is ± 1 SEM over the trials, with a negative value showing that the protein favors state j relative to solution. Populations are means over the three trials. The ponatinib row is the surface-bound site of 3ZOS. A pair is listed only where all four populations exceed 10^{-3} in every trial.

PDB	Inhibitor	State i	State j	$P(i)_{\mathrm{soln}}$	$P(j)_{\mathrm{soln}}$	$P(i)_{\mathrm{prot}}$	$P(j)_{\mathrm{prot}}$	$\Delta\Delta G$ (kcal/mol)	$\pm$SEM
3PYY	Imatinib	STI+1	STI+2	0.588	0.380	0.004	0.996	−3.54	0.09
4AGC	Axitinib	AXI01	AXI02	0.810	0.187	0.988	0.012	+1.75	0.05
3UE4	Bosutinib	DB8+1	DB8+2	0.888	0.057	0.566	0.389	−1.40	0.01
3ZOS	Ponatinib	0LI+1	0LI+2	0.589	0.375	0.181	0.715	−1.08	0.05
4AG8	Axitinib	AXI01	AXI02	0.810	0.187	0.961	0.039	+1.02	0.01
4ZAU	Osimertinib	YY3+1	YY3+2	0.974	0.004	0.985	0.003	+0.14	0.14

Table S6: Solution protonation states of the inhibitors compared with literature reference pK_a values. Reported here are the listed ligand charge state in solution (q_{soln}) and its population (P), both from Table S2; the pK_a obtained from the Epik state penalties through Equations S2–S4; the deviation from the literature reference (Δ, calculated minus reference); and the moiety whose protonation state changes (Group). Calculated values are given to two decimal places so that Δ is reproducible from the columns, which is finer than the accuracy of the comparison. The dominant solution charge state is shown except for crizotinib, for which both +1 and +2 states are listed. Entries marked n.a. indicate that the corresponding charge transition is absent from the enumerated set or that no literature reference value was located.

Inhibitor	Group	q_{soln}	P	pK_a(calc)	pK_a(exp)	Δ
Afatinib	dimethylamine	+1	0.992	9.49	8.02[a]	+1.47
Alectinib	piperidine/morpholine	+1	0.561	7.51	7.05[a]	+0.46
Axitinib	pyridine	+0	0.997	4.62	4.80[a]	−0.18
Bosutinib	n.a.	+1	0.945	8.63	n.a.	n.a.
Ceritinib	piperidine	+1	0.999	10.31	9.70[a]	+0.61
Cobimetinib	n.a.	+1	0.986	9.25	n.a.	n.a.
Crizotinib	piperidine	+1	0.380	9.31	9.40[a]	−0.09
Crizotinib	aminopyridine	+2	0.615	7.62	5.60[a]	+2.02
Gefitinib	morpholine	+0	0.700	7.03	7.20[a]	−0.17
Imatinib	piperazine	+1	0.968	8.87	8.07[b]	+0.80
Lapatinib	n.a.	+1	0.949	8.67	n.a.	n.a.
Lenvatinib	quinoline	+0	1.000	n.a.	5.05[a]	n.a.
Nilotinib	imidazole	+0	0.907	6.41	5.40[c]	+1.01
Osimertinib	n.a.	+1	0.979	9.08	n.a.	n.a.
Palbociclib	piperazine	+1	0.975	9.00	7.40[a]	+1.60
Ponatinib	piperazine	+1	0.963	8.87	7.80[a]	+1.07
Sorafenib	n.a.	+0	0.996	5.05	n.a.	n.a.
Sunitinib	diethylamine	+1	0.987	9.28	8.95[a]	+0.33
Tofacitinib	n.a.	+0	0.971	5.88	n.a.	n.a.

Inhibitor	Group	q_{soln}	P	pK_{a}(calc)	pK_{a}(exp)	Δ

[a] Prescribing information or product monograph for the approved product. The afatinib value is reported as 8.02 ± 0.01 in water at 25 °C and ionic strength 0.05 M; the other regulatory values are quoted without uncertainties. The lenvatinib and sunitinib values are reported for the mesylate and malate salts respectively. [b] Macroconstant from NMR–pH and pH-potentiometric titration.[9] [c] Value reported by the source as estimated rather than measured. Where a source reports a pK_{a} without assigning it to a group, the group named here is the basic center identified by inspection of the structure.

References

(1) Honig, B.; Sharp, K.; Yang, A. S. Macroscopic models of aqueous solutions: biological and chemical applications. The Journal of Physical Chemistry **1993**, 97, 1101–1109.

(2) Baker, N. A. Improving implicit solvent simulations: a Poisson-centric view. Current Opinion in Structural Biology **2005**, 15, 137–143.

(3) Warwicker, J.; Watson, H. Calculation of the electric potential in the active site cleft due to -helix dipoles. Journal of Molecular Biology **1982**, 157, 671–679.

(4) Rocchia, W.; Alexov, E.; Honig, B. Extending the Applicability of the Nonlinear PoissonBoltzmann Equation: Multiple Dielectric Constants and Multivalent Ions. The Journal of Physical Chemistry B **2001**, 105, 6754–6754.

(5) Cornell, W. D.; Cieplak, P.; Bayly, C. I.; Gould, I. R.; Merz, K. M.; Ferguson, D. M.; Spellmeyer, D. C.; Fox, T.; Caldwell, J. W.; Kollman, P. A. A Second Generation Force Field for the Simulation of Proteins, Nucleic Acids, and Organic Molecules. Journal of the American Chemical Society **1995**, 117, 5179–5197.

(6) Gunner, M. R.; Zhu, X.; Klein, M. C. MCCE analysis of the pKas of introduced buried acids and bases in staphylococcal nuclease. Proteins: Structure, Function, and Bioinformatics **2011**, 79, 3306–3319.

(7) Song, Y. F.; Mao, J. J.; Gunner, M. R. MCCE2: Improving Protein pKa Calculations with Extensive Side Chain Rotamer Sampling. Journal of Computational Chemistry **2009**, 30, 2231–2247, Type: Journal Article.

(8) Levy, R. M.; Zhang, L. Y.; Gallicchio, E.; Felts, A. K. On the Nonpolar Hydration Free Energy of Proteins: Surface Area and Continuum Solvent Models for the SoluteSolvent Interaction Energy. Journal of the American Chemical Society **2003**, 125, 9523–9530.

(9) Szakács, Z.; Béni, S.; Varga, Z.; Örfi, L.; Kéri, G.; Noszál, B. Acid- base profiling of imatinib (gleevec) and its fragments. Journal of medicinal chemistry **2005**, 48, 249–255.
